# Dendritic Cell‐Hitchhiking In Vivo for Vaccine Delivery to Lymph Nodes

**DOI:** 10.1002/advs.202402199

**Published:** 2024-07-04

**Authors:** Lei Zhou, Ling Zhao, Mengyao Wang, Xu Qi, Xin Zhang, Qingying Song, Dayu Xue, Meihua Mao, Zhenzhong Zhang, Jinjin Shi, Pilei Si, Junjie Liu

**Affiliations:** ^1^ School of Pharmaceutical Sciences Zhengzhou University Zhengzhou 450001 China; ^2^ Key Laboratory of Targeting Therapy and Diagnosis for Critical Diseases Zhengzhou 450001 China; ^3^ Department of Breast Surgery, Henan Provincial People's Hospital People's Hospital of Zhengzhou University People's Hospital of Henan University Zhengzhou Henan 450003 China

**Keywords:** dendritic cell‐hitchhiking, lymph node targeting, vaccine delivery

## Abstract

Therapeutic cancer vaccines are among the first FDA‐approved cancer immunotherapies. Among them, it remains a major challenge to achieve robust lymph‐node (LN) accumulation. However, delivering cargo into LN is difficult owing to the unique structure of the lymphatics, and clinical responses have been largely disappointing. Herein, inspired by the Migrated‐DCs homing from the periphery to the LNs, an injectable hydrogel‐based polypeptide vaccine system is described for enhancing immunostimulatory efficacy, which could form a local niche of vaccine “hitchhiking” on DCs. The OVA peptide modified by lipophilic DSPE domains in the hydrogel is spontaneously inserted into the cell membrane to achieve “antigen anchoring” on DCs in vivo. Overall, OVA peptide achieves active access LNs through recruiting and “hitchhiking” subcutaneous Migrated‐DCs. Remarkably, it is demonstrated that the composite hydrogel enhances LNs targeting efficacy by approximately six‐fold compared to free OVA peptide. Then, OVA peptide can be removed from the cell surface under a typical acidic microenvironment within the LNs, further share them with LN‐resident APCs via the “One‐to‐Many” strategy (One Migrated‐DC corresponding to Many LN‐resident APCs), thereby activating powerful immune stimulation. Moreover, the hydrogel vaccine exhibits significant tumor growth inhibition in melanoma and inhibits pulmonary metastatic nodule formation.

## Graphical abstract

A major challenge in vaccine delivery is to achieve robust lymph node accumulation. Herein, this paper describes an injectable hydrogel‐based polypeptide vaccine system for enhancing immunostimulatory efficacy. Remarkably, the composite hydrogel enhances lymph node targeting by approximately six‐fold compared to free OVA peptide, and significantly inhibits melanoma growth as well as pulmonary metastatic nodule formation.

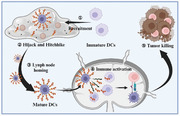

## 1 Introduction

Therapeutic cancer vaccines, characterized by high specificity, low toxicity, and few adverse reactions, are among the first FDA‐approved cancer immunotherapies.^[^ ^1^, ^2^, ^3^ ^]^ They are promising options for replacing or assisting traditional therapies to enhance efficacy.^[^ ^4^, ^5^ ^]^ Despite multiple clinical trials that have demonstrated the safety and immunogenicity of cancer vaccines, clinical responses have been largely disappointing.^[^ ^6^, ^7^ ^]^ Among them, lymph nodes (LNs) are the critical sites to initiate adaptive immune responses.^[^ ^8^, ^9^ ^]^ However, interstitial administration (for example, intramuscular, subcutaneous, and intradermal administration), the main mode of administration routes for current vaccines clinical trials, is relatively poor targeting ability to LNs because the majority of vaccines still remain in the peripheral injection sites owing to the complex delivery barriers generated by the dynamic physiological environment within the interstitium.^[^ ^10^, ^11^, ^12^ ^]^ Meanwhile, the clearance of vaccines via blood capillaries and potential endocytosis by other cell types would also reduce the delivery efficacy of vaccines toward LNs.^[^ ^12^, ^13^ ^]^ Therefore, an effective vaccine delivery strategy to enhance LNs targeting and clinical efficacy is highly desired.

Intranodal injection provides the most direct and efficient vaccine delivery strategy to LNs, a lower dose might be administered to achieve a strong immune response.^[^ ^14^, ^15^ ^]^ However, clinical LN localization is difficult even with the help of ultrasound or the nontoxic tracer dye, and the highly delicate structure of LNs and cytokine gradients inside might be irreparably damaged by direct injection.^[^ ^16^, ^17^ ^]^ These issues make intranodal injection impractical. Recently, nano‐delivery systems offer the possibility of effective delivery to LNs, which mostly traffic to LNs via discontinuous peripheral lymphatic vessels.^[^ ^18^, ^19^ ^]^ In previous study, the physicochemical characteristics (for example, size, charge, and deformability) of nano‐vaccines can be utilized to enhance the targeting efficiency of their transport from peripheral tissues to lymphatic organs via the lymphatic system owing to the potential of nanoparticles to prolong circulation times of infused agents or enhance the efficiency of transport across different barriers, to leverage specific physiological structures and pathways to improve LNs targeting or clearance pathways.^[^ ^20^, ^21^, ^22^ ^]^ However, nanoparticles could only passively drain into the lymphatic vessels since LN is an one direction transit network without a central drive force, which leads to the limitation of the targeted delivery efficiency of LNs.^[^ ^15^ ^]^ Moreover, the inner cortical region still contains narrow conduits of 3–5 nm in diameter even when entering the LNs through lymphatic vessels, which only allow the entry of small molecules with molecular weights less than 70 kDa or very small particles (2–5 nm) to penetrate into the deep LNs.^[^ ^23^, ^24^ ^]^ In this case, despite extensive efforts, none of these agents have received FDA approval for tumor immunotherapy due to limited efficacy. Thus, it would be of great clinical significance to develop a platform of antigens active delivery that facilitates accumulation in LNs.

**Scheme 1:**
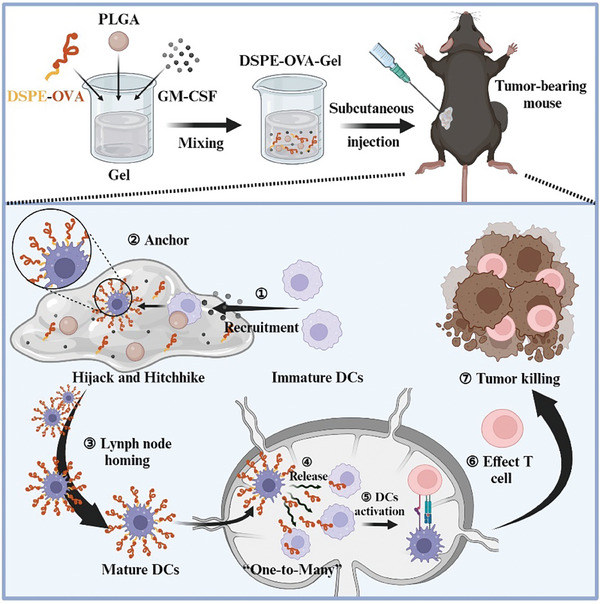
Schematic diagram of the hydrogel‐based vaccine to enhance tumor immunotherapy by DCs “hitchhiking”. First, the schematic diagram shows the preparation of DSPE‐OVA‐Gel, which consists of pH‐responsive DSPE‐OVA, porous PLGA microspheres, and GM‐CSF. DSPE‐OVA‐Gel solution is cross‐linked with the cations present to form hydrogel after subcutaneous injection in mice. Then, GM‐CSF recruits sufficient Migrated‐DCs to hydrogel, and porous PLGA microspheres with rough surfaces provide attachment sites for DCs. When the Migrated‐DCs are recruited and resided in hydrogel, the model antigen OVA modified by DSPE is spontaneously inserted into the DCs membrane in hydrogel based on its high membrane binding affinity to achieve “OVA anchoring” on DCs in vivo. Finally, the OVA‐bearing DCs migrate to LNs, which induce sufficient effector *T*‐cells via the one Migrated‐DC corresponding to many LN‐resident APCs strategy under the LN typical acidic microenvironment.

Dendritic cells (DCs), known as the most potent antigen‐presenting cells (APCs) so far, spread throughout the tissues to scan antigens.^[^ ^25^, ^26^ ^]^ Importantly, adaptive immune responses depend on the ability of mature DCs homing to LNs for further antigen presentation.^[^ ^27^ ^]^ Inspired by the Migrated‐DCs homing from the periphery to the LNs, in mind, we prepared DSPE‐Ovalbumin peptide (DSPE‐OVA) ——via a single‐step cross‐linking chemistry of FDA‐approved DSPE‐PEG and the model antigen OVA peptide (OVA)——that would achieve accumulation in lymphoid organs through “hitchhiking” with endogenous Migrated‐DCs (**Scheme** **1**). To enhance the efficiency of antigen “hijacking” by Migrated‐DCs, we prepared an injectable DSPE‐OVA hydrogel (DSPE‐OVA‐Gel) as a local niche for vaccine “hitchhiking” on DCs, which consisted of pH‐responsive DSPE‐OVA, granulocyte‐macrophage colony‐stimulating factor (GM‐CSF) to recruit and activate Migrated‐DCs, and porous PLGA microspheres with rough surfaces to provide attachment sites for DCs. When the DCs were recruited and resided in the hydrogel, DSPE‐OVA could efficiently insert into the DC membrane based on high affinity between the lipophilic DSPE tail and cytomembrane to improve anchoring efficiency. This process could achieve higher loading of OVA without damaging the membrane and disrupting the cellular signaling of DCs. Given that mature DCs have natural LNs homing ability, OVA‐bearing DCs could accurately and efficiently target to LNs. Finally, OVA could be removed from the cell surface under typical acidic microenvironment within the LNs, further shared them with LN‐resident APCs via the “One‐to‐Many” strategy (One Migrated‐DC corresponding to Many LN‐resident APCs). Our study demonstrates that this injectable composite hydrogel vaccine significantly enhances antitumor immunity by achieving the delivery dose to LNs and triggering potent adaptive immune signaling.

## 2 Results

### 2.1 Preparation and Characterization of DSPE‐OVA‐Gel

Exploiting DCs as an antigen transporter, we proposed that antigens modified with lipophilic DSPE domains would load on DCs through in situ anchoring for active LN transport. In order to accelerate this process, we constructed a composite hydrogel containing GM‐CSF, DSPE‐OVA, and porous PLGA microspheres.

**Figure 1:**
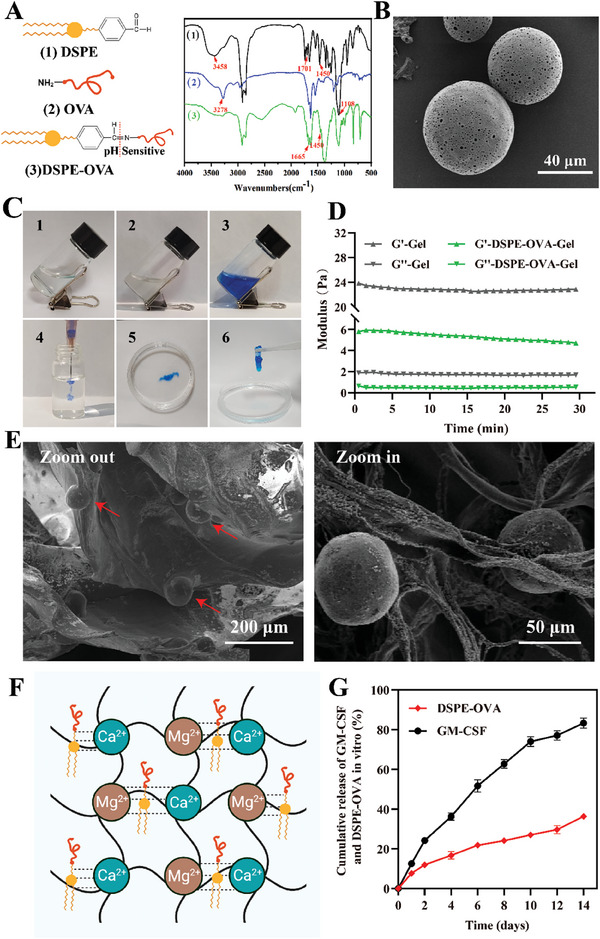
Preparation and characterization of DSPE‐OVA‐Gel vaccine. A) FT‐IR spectra of 1) DSPE, 2) OVA and 3) DSPE‐OVA. 1) The characteristic C═O peak of aromatic aldehydes at 1701 cm^−1^ and C═C stretching vibration peaks of benzene ring structure at 1450 cm^−1^ are present; 2) The characteristic peaks of OVA were at 3278 cm^−1^ representing NH functional groups (primary amine); 3) A new absorption peak at 1665 cm^−1^ was attributed to the absorption of ─C═N─ in DSPE–OVA. B) Representative TEM image of PLGA (scale bar: 40 µm). C) The photos of 1) the sodium alginate solution, 2) DSPE‐OVA‐Gel solution and 3,4) MB‐containing the DSPE‐OVA‐Gel solution. Gel formation of D**S**PE‐OVA‐Gel 5,6) by adding into the cation mixed solution of 1.8 × 10^−3^ mol L^−1^ CaCl_2_ and 1.5 × 10^−3^ mol L^−1^ MgCl_2_. D) The rheological (storage modulus G′ and loss modulus G″) properties of the hydrogel with/without DSPE‐OVA, PLGA and GM‐CSF. E) The zoom‐out (left) and zoom‐in (right) SEM images of D**S**PE‐OVA‐Gel (the scale bar is 200 µm in the left image and 50 µm in the right enlarged image). F) Schematic diagram of the D**S**PE‐OVA‐Gel formation. G) Cumulative release of GM‐CSF and D**S**PE‐OVA from D**S**PE‐OVA‐Gel in vitro (*n* = 3 independent experiments). The results are shown as the mean ± standard deviation.

First, based on the physiological feature that LNs are maintained at a mean pH of ≈6.4,^[^ ^28^ ^]^ the copolymer DSPE–OVA was synthesized via the reversible Schiff base between the benzaldehyde at DSPE and amino groups on OVA.^[^ ^29^ ^]^ The synthesis processes are shown in Figure S1 (Supporting Information) and the FT‐IR spectra are shown in **Figure** **1**A. After conjugation between DSPE and OVA, a new absorption peak at 1665 cm^−1^ appeared, which was attributed to the absorption of ─C═N─ in DSPE–OVA. Additionally, the absorption peak at 1450 cm^−1^ on the benzene ring structure was still preserved indicating the successfully conjugation of DSPE with OVA to form DSPE‐OVA. Then, we further demonstrated that the DSPE‐OVA remained stable at physiological pH; however, OVA detached from DSPE‐OVA under an acidic microenvironment within the LNs, which was acid‐sensitive (Figure S2, Supporting Information).

Furthermore, we fabricated porous PLGA microspheres (PLGA) by double emulsion solvent evaporation method to enhance the anchoring efficiency of DCs. The morphology of PLGA was assessed by scanning electron microscope (SEM), which possessed round shape with diameter ≈60 µm, and the porous outer surface could be observed (Figure 1B). To create the hydrogel as a local niche for vaccine “hitchhiking” on DCs, sodium alginate hydrogel is chosen due to low cost, commercial availability, and excellent biocompatibility.^[^ ^30^ ^]^ The sodium alginate hydrogel (Gel) was formed using the standard sodium‐to‐calcium/magnesium ion exchange method. Considering the physiological concentrations of multivalent cations, subcutaneous injection of 2 wt.% sodium alginate solution is able to form a hydrogel, which provides great convenience for the injectability and drug loading. In order to observe whether the addition of DSPE‐OVA, PLGA and GM‐CSF influenced the gelation, methylene blue (MB) dye was added to 2 wt.% Gel loaded with DSPE‐OVA (10 mg mL^−1^), PLGA (4%) and GM‐CSF (10 µg mL^−1^) (DSPE‐OVA‐Gel, Figure 1C (1, 2, 3)), which was conducive to the observation of the gelation. At this stage, the DSPE‐OVA‐Gel in the non‐gel state remained injectable (Figure 1C (4)). Then, the DSPE‐OVA‐Gel solution was added into the cation mixed solution of 1.8 × 10^−3^ mol L^−1^ CaCl_2_ and 1.5 × 10^−3^ mol L^−1^ MgCl_2_, which simulated the physiological concentrations of Ca^2+^ and Mg^2+^. Within 2 h, DSPE‐OVA‐Gel was formed the gelation at room temperature (Figure 1C (5, 6)). Meanwhile, the maximum loading capacity of DSPE‐OVA in the Gel was obtained when the 2 wt.% Gel loaded with 40 mg mL^−1^ DSPE‐OVA (Figure S3, Supporting Information), and the G′ (storage modulus) was still higher than G″ (loss modulus) with a high modulus, indicating that the formation of the DSPE‐OVA‐Gel was basically unaffected (Figure 1D).

Furthermore, the 3D network structure of DSPE‐OVA‐Gel could be observed by the SEM as shown in Figure 1E. The PLGA could be observed to adhere in the gel network by magnification. Since the synthesized DSPE‐OVA and GM‐CSF are macromolecules, they are difficult to observe in the SEM images. In order to achieve the recruitment of DCs into the hydrogel before DSPE‐OVA leakage, in our design, release rates of GM‐CSF and DSPE‐OVA from hydrogel were significantly different due to their different sizes and electrostatic attraction (negatively charged DSPE‐OVA), which was conducive to the stable existence of DSPE‐OVA in the hydrogel for a longer period of time (Figure 1F). As indicated in Figure 1G and Figure S4 (Supporting Information), more than half of GM‐CSF was released within a week, and only 16.6% remained after 14 days, while most of DSPE‐OVA still remained in the DSPE‐OVA‐Gel, whose ratios were 77.1% at day 7 and 63.4% at day 14. These were consistent with our previous expectations, and laid the foundation for the delivery system application in vivo.

### 2.2 DCs Recruitment by the DSPE‐OVA‐Gel In Vitro and In Vivo

**Figure 2:**
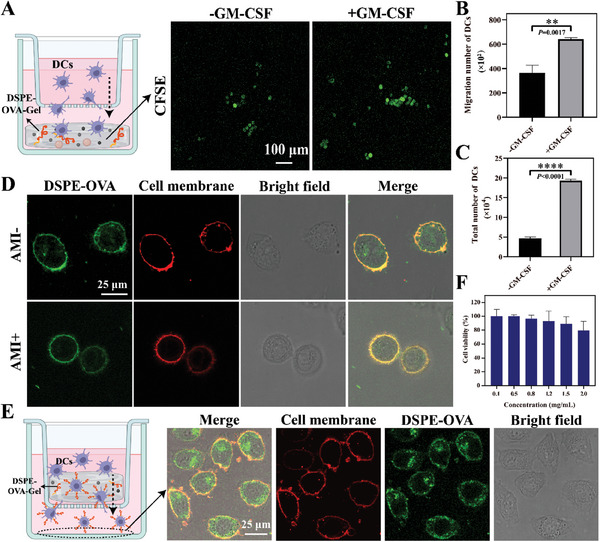
DCs recruitment and OVA anchoring via DSPE‐OVA‐Gel. A) Schematics for transwell model of DCs recruitment. Representative CLSM images of DCs (green) recruited into the hydrogel after 24 h of coculture (scale bar: 100 µm). B) Statistical analyses for the number of DCs recruited to the hydrogel (*n* = 3 independent experiments). C) Total number of DCs in DSPE‐OVA‐Gel loaded with GM‐CSF on 5th day after subcutaneous injection. D) Representative CLSM images of DCs loaded with DSPE‐OVA in the presence/absence of preincubation with amiloride (DSPE‐OVA: green; cell membrane: red, scale bar: 25 µm). E) The ability of DCs to load DSPE‐OVA in the hydrogel was investigated by transwell coculture system (DSPE‐OVA: green; cell membrane: red, scale bar: 25 µm). A schematic diagram of transwell coculture system is shown on the left. F) The cytotoxicity of DCs treated with DSPE‐OVA was assessed in CCK‐8 assays (24 h), expressed as a percentage of normal DCs (*n* = 3 independent experiments). The results are shown as the mean ± standard deviation. Statistical analyses were performed via the two‐sided Student's *t*‐test, ^**^ *p* < 0.01; ^****^ *p* < 0.0001.

Several studies have shown that GM‐CSF plays an important role in DCs recruitment and maturation.^[^ ^31^, ^32^, ^33^ ^]^ To observe the recruitment of DCs into DSPE‐OVA‐Gel, the modified transwell migration assay was utilized to assess, and DSPE‐OVA‐Gel without GM‐CSF was selected as control. Then, we labeled DCs with carboxy‐fluorescein diacetate succinimidyl ester (CFSE) dye, which allowed to quantify DCs migrating to the hydrogel by confocal laser scanning microscope (CLSM). As shown in **Figure** **2**A,B, compared with control group, the DSPE‐OVA‐Gel loaded with GM‐CSF could recruit about twice as many DCs into the hydrogel. Moreover, in order to further investigate the recruitment ability of DCs in vivo, the DSPE‐OVA‐Gel was subcutaneously injected to form hydrogels, which were taken out subsequently for DCs counting and hematoxylin and eosin (H&E) staining. The DSPE‐OVA‐Gel loaded with GM‐CSF significantly recruited more DCs than control, and increased in a time‐dependent manner in vivo (Figure 2C; Figure S5a, Supporting Information). Meanwhile, the total number of cells increased via H&E staining images (Figure S5b, Supporting Information). In addition, our results also showed that the PLGA in DSPE‐OVA‐Gel could prolong the residence time of DCs in gel (Figure S6, Supporting Information), which contributed to the further anchoring of DSPE‐OVA on the DCs surface.

### 2.3 DSPE‐OVA Anchors on DCs Within the DSPE‐OVA‐Gel

As illustrated in Scheme 1, our goal is to facilitate the anchoring of DSPE‐OVA on DCs. Based on DCs being recruited and remaining in DSPE‐OVA‐Gel, DSPE‐OVA with a lipophilic DSPE tail could be inserted into the phospholipid bilayer of the cell membrane and loaded on the cell surface.^[^ ^34^, ^35^, ^36^ ^]^ However, DCs possess the natural phagocytic behavior, and some studies have shown that the uptake mechanism of OVA is mainly via micropinocytosis.^[^ ^37^, ^38^ ^]^ Then, DCs were pre‐incubated with amiloride (AMI, 100 µg mL^−1^, 1 h), a micropinocytosis inhibitor, and the distribution of DSPE‐OVA was analyzed by CLSM to distinguish between uptake and membrane anchoring. As indicated in Figure 2D, when cells were co‐incubated with DSPE‐OVA for 2 h, there was a clear co‐localization between DSPE‐OVA (green) and the cell membrane (red). In the presence of AMI, OVA was significantly reduced within the DCs but not on the cell membrane surface, suggesting that OVA could insert into the cell membrane via the hydrophobic end of the DSPE. Then, we used transwell chambers to mimic the in vivo state of the DSPE‐OVA‐Gel, and determined whether the DSPE‐OVA anchored to the cell membrane during DCs migration inside the hydrogel. Analyses of the lower compartment showed that the DCs membrane was loaded with large amounts of DSPE‐OVA in DSPE‐OVA‐Gel (Figure 2E). Meanwhile, the maximum concentration of DSPE‐OVA anchored to the cell membrane was examined by flow cytometry. As shown in Figure S7 (Supporting Information), the fluorescence intensity no longer increased when the added drug concentration was 400 µg mL^−1^, at which point the maximum concentration of DSPE‐OVA was administered. The calculated results showed that the 3 × 10^6^ DCs could load ≈175.12 µg DSPE‐OVA and release 143.75 µg OVA in the simulated LN microenvironment (Figures S4b and S8, Supporting Information). Meanwhile, this loading process did not affect their viability (Figure 2F) and migration ability (Figure S9, Supporting Information) of DCs. These were consistent with our expectations. Collectively, these data indicated that the DSPE‐OVA‐Gel successfully recruited Migrated‐DCs and the antigens could effectively “hitchhike” on DCs without affecting their viability, which laid the foundation for further LNs delivery as well as immunological activation of the vaccine in vivo.

### 2.4 Exploiting the “Hitchhike” for Potent Lymph Node Delivery

**Figure 3:**
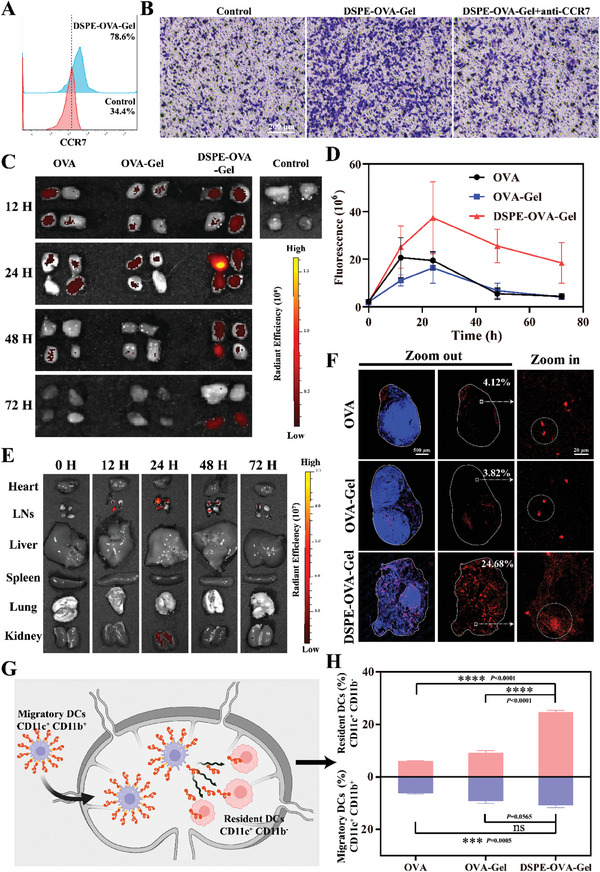
Exploiting the “hitchhike” for potent lymph node delivery. A) Flow cytometry for surface expression of CCR7 in DC2.4 cells after the hydrogel treatment. B) CCL21‐induced migration of DC2.4 cell was assessed by transwell assay in vitro. CCR7‐neutralizing antibody (20 µg mL^−1^) was used to block the chemokine receptors CCR7 on the cell surface of DC2.4 cells. (scale bar: 200 µm) C) IVIS fluorescence imaging of the isolated inguinal and axillary LNs from C57BL/6 mice at different time points after vaccine administration. D) The corresponding quantitative fluorescence intensity in LN drainage over time (*n* = 3 independent experiments). E) The heart, LNs, liver, spleen, lung, and kidney were excised from mice treated with DSPE‐OVA‐Gel. IVIS imaging and DSPE‐OVA‐Gel distribution among these tissues at the indicated times post‐treatment. F) Antigen accumulations within the LNs 24 h after administration. The nucleus and antigens are labeled by DAPI (blue) and TAMRA (red), respectively (scale bar of left and middle images, 500 µm; scale bar of right enlarged images, 20 µm). G) Schematic diagram of the DSPE‐OVA‐Gel potential mechanism for LNs delivery. H) The tendency of delivery pathways based on the proportion of Resident‐DCs (CD11c^+^ CD11b^−^) and Migratory‐DCs (CD11c^+^ CD11b^+^) within the LNs (*n* = 3 independent experiments). The results are shown as the mean ± standard deviation. Statistical analyses were performed via ordinary one‐way analyses of variance (ANOVA) with Tukey's multiple comparisons test, no significant (ns): *p* > 0.05; ^***^ *p* < 0.001; ^****^ *p* < 0.0001.

After DCs capturing antigen and developing into mature in the periphery, the signaling pathway triggered by the engagement of the lymphoid chemokines CCL19 and CCL21 with their cognate receptor CCR7 has a central role in Migrated‐DCs to draining LNs via afferent lymphatics, and promotes the development of immune response.^[^ ^39^, ^40^ ^]^ To elucidate the homing effect of this system, we selected the main components in the hydrogel vaccine (DSPE‐OVA, PLGA, and GM‐CSF) to investigate the main factors inducing DCs maturation. As shown in Figure S10 (Supporting Information), both GM‐CSF and DSPE‐OVA obviously improved the maturation of DCs, while the effect of PLGA itself on DCs maturation is negligible. In addition, the highest proportion of mature DCs was observed under the coexistence of three components. Furthermore, DCs treated with DSPE‐OVA‐Gel upregulate the expression of CCR7 and promote migration to the lymphoid chemokines CCL21 (**Figure** **3**A). Conversely, when CCR7 activation was blocked with CCR7‐neutralizing antibody (20 µg mL^−1^), the migration ability of DCs significantly decreased (Figure 3B).

To investigate whether DCs loaded with OVA could accurately and efficiently homing to LNs, the preparations (5‐TAMRA labeled OVA) were provided subcutaneously in C57BL/6 mice, and the draining LNs in inguinal and axillary were excised at 0, 12, 24, 48, and 72 h for fluorescence imaging after injection. As shown in Figure 3C,D, among all groups, the fluorescence intensity in the DSPE‐OVA‐Gel group showed the most significant increase and the strongest at each time point. Strikingly, only DSPE‐OVA‐Gel group still retained fluorescence in the LNs at 72 h, indicating that the LN targeting of DSPE‐OVA‐Gel was excellent. In addition, the fluorescence intensity of free OVA and DSPE‐OVA‐Gel successively peaked at 12 and 24 h after administration, further supporting the time‐sequence variations between passive diffusion and “hitchhiking” pathways. The OVA‐Gel group showed the weakest fluorescence at 12 h, which might be due to the fact that hydrogel encapsulation limited the passive diffusion of OVA, and did not have the ability of “hitchhike” on DCs due to the lack of DSPE modification. Therefore, we conducted the subsequent biodistribution on DSPE‐OVA‐Gel in vivo. Consistent with the above results, LN fluorescence was observed at 12 h, with the peak accumulation occurring at 24 h. At the same time, its fluorescence mainly gathered in LNs while other tissues could be ignored (Figure 3E).

Then, we further dissected the LNs and analyzed the distribution of OVA. From the whole LNs image, DSPE‐OVA from DSPE‐OVA‐Gel accumulated within the LNs, with evidently larger antigen‐infiltrated areas (24.68%), compared with those with free OVA (4.12%) and OVA‐Gel (3.82%). These findings were in line with our previous study. Meanwhile, DSPE‐OVA‐Gel group was distributed far more deeply and uniformly into the tissue, which differed from the small amount of aggregated fluorescence in the free OVA and OVA‐Gel groups (Figure 3F). This distribution pattern was considered to be due to the OVA release from Migrated‐DCs in response to the acidic LNs microenvironment and reuptake by LN‐resident APCs. It was also further confirmed at the cellular level (Figure S11a, Supporting Information).

We further validated the subcutaneous loading and “hitchhiking” pathways of DSPE‐OVA‐Gel for LN‐targeting based on the above findings. Within the LNs, the Resident‐DCs (CD11c^+^ CD11b^−^) can only uptake antigens via direct LN delivery from DSPE‐OVA‐Gel (release in acid LNs microenvironment), whereas the Migrated‐DCs (CD11c^+^ CD11b^+^) can uptake antigens subcutaneously (Figure 3G).^[^ ^20^ ^]^ Thus, the origin of DCs in the LNs was determined to analyze the LN‐targeting of DSPE‐OVA‐Gel. As shown in Figure 3H, DSPE‐OVA‐Gel was found to induce the highest number of antigen‐presenting DCs, among which 11.07% were Migrated‐DCs recruited from the peripheral tissues and 24.8% were Resident‐DCs. These dramatic increases in both indicated that the “One‐to‐Many” strategy (One Migrated‐DC corresponding to Many LN‐resident APCs) of DSPE‐OVA‐Gel allowed OVA release and re‐delivery in LNs. In the OVA‐Gel group, most DCs (Migratory‐DCs) were contributed by antigen uptake and subsequent homing alone, which was not sufficient to re‐activate Resident‐DCs. Additionally, the re‐delivery of DSPE‐OVA‐Gel was also confirmed via simulating acid response release and re‐incubation with Resident‐DCs in vitro (Figure S11b, Supporting Information). Taken together, these data indicated that we developed an efficient system, which induced potent antigen depots at the administration sites to recruit and “hitchhike” with Migrated‐DCs for the active targeting as well as accumulation of antigens in LNs. Once in the LNs, the antigens were released from the Migrated‐DCs under acidic microenvironment and taken up by resident APCs.

### 2.5 DSPE‐OVA‐Gel Buttresses Robust Immune Responses In Vivo

**Figure 4:**
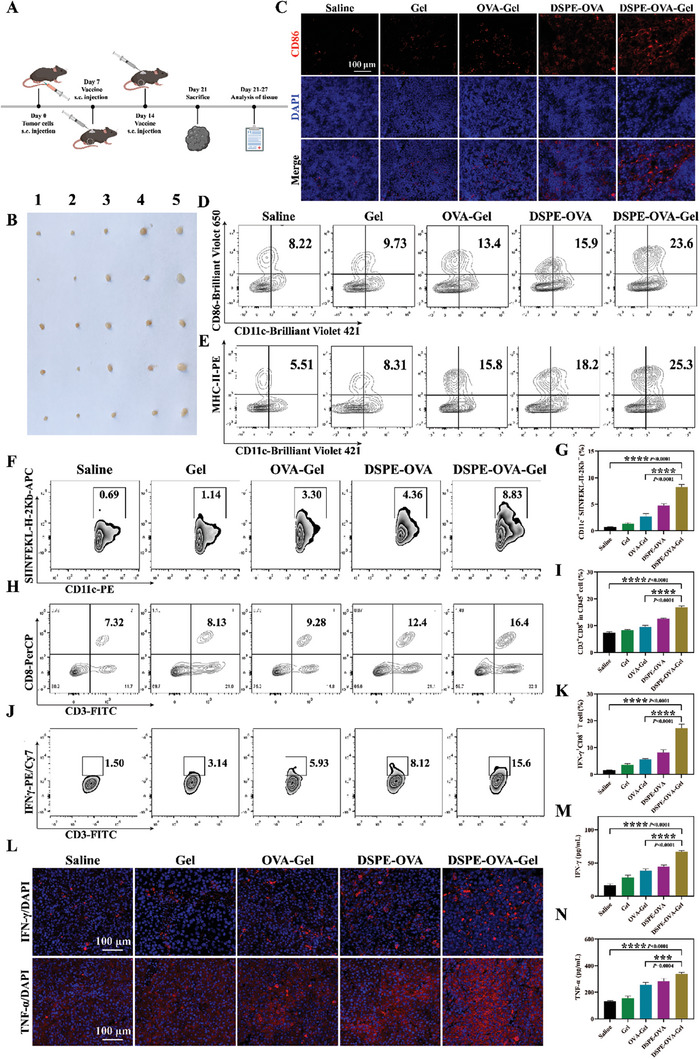
DSPE‐OVA‐Gel buttresses robust immune responses in vivo. A) The experimental design to evaluate the immune responses by the DSPE‐OVA‐Gel. B) Enlargement of LNs after vaccination (1: Saline, 2: Gel, 3: OVA‐Gel, 4: DSPE‐OVA, 5: DSPE‐OVA‐Gel, *n* = 5 independent experiments). C) Immuno‐fluorescence images of CD86 in LNs (CD86: red; DAPI: blue, scale bar: 100 µm). Representative flow cytometric analyses of CD11c^+^ CD86^+^ cells D), CD11c^+^ MHC‐II^+^ cells E), CD11c^+^ SIINFEKL‐H‐2Kb^+^ cells (gated on CD11c^+^) F) and CD3^+^ CD8^+^ *T*‐cells (gate on CD45^+^) H) infiltrated in LNs after receiving different treatments in C57BL/6 mice (*n* = 3 independent experiments). The relative quantification of CD11c^+^ SIINFEKL‐H‐2Kb^+^ cells G) and CD8^+^ *T*‐cells I) infiltrated in LNs. Flow cytometry analyses J) and relative quantification K) of IFN‐γ^+^ CD8^+^ *T*‐cells (gate on CD45^+^ CD3^+^) in peripheral blood. L) Representative immuno‐fluorescence staining for IFN‐γ and TNF‐α in tumor tissues (IFN‐γ/TNF‐α: red; Nucleus: blue, scale bar: 100 µm, *n* = 3 independent experiments). Serum levels of IFN‐γ M) and TNF‐α N) were measured by ELISA (*n* = 3 independent experiments). The results are shown as the mean ± standard deviation. Statistical analyses were performed via ordinary one‐way ANOVA with Tukey's multiple comparisons test, ^***^ *p* < 0.001; ^****^ *p* < 0.0001.

Encouraged by the excellent LNs‐targeting of antigens in vivo, we utilized a syngeneic murine model of melanoma with B16‐F10 cells expressing ovalbumin (B16‐OVA) to further evaluate the immunization efficacy of DSPE‐OVA‐Gel. After subcutaneous inoculation of 2 × 10^6^ B16‐OVA cells, the indicated formulations were subcutaneously administrated into the back of mice on days 7 and 14 to test the immune‐potentiation effect. At day 21, the LNs, serum, and tumor of mice were collected for immune response detection (**Figure** **4**A).

First, since LNs are essential for the activation of immune responses against tumors, as demonstrated in Figure 4B, inguinal LNs adjacent to the injection site were clearly enlarged, which was considered to be due to a significant change in LNs architecture, lymphocyte proliferation, and expansion of LNs stroma occur following antigen‐mediated activation of intranodal immune responses. Once the antigen being captured by resident APCs in LNs, efficient innate immune response activation and antigen presentation are important for vaccines to trigger robust antitumor immunity. Then, we utilized DCs as representative APCs to examine the maturation and antigen presentation by flow cytometric analyses. Compared with the OVA‐Gel group, the ratio of DCs maturation in the LNs was markedly increased in the DSPE‐OVA‐Gel groups (Figure 4C,D). As indicated by a rapid increase in the proportion of CD11c^+^ MHC‐II^+^ (Figure 4E) and CD11c^+^ SIINFEKL/H‐2Kb^+^ positive cells (Figure 4F,G), the hydrogel vaccine could effectively activate antigen‐specific immunity. Meanwhile, the proportion of CD4^+^ (Figure S12a,b, Supporting Information) and CD8^+^ (Figure 4H,I) *T*‐cells in LNs were also significantly increased in the hydrogel vaccine, which was consistent with our previous expectations. However, in DSPE‐OVA group without Gel, a slight level of DCs with maturation and antigen presentation was detected. This might be related to the rapid degradation of peptides in vivo, which illustrated the importance of hydrogel protection and the indispensability of GM‐CSF as well as PLGA in the hydrogel. In addition, we proved earlier that PLGA could enhance the resident function of DCs in hydrogel without promoting DCs maturation. To further verify the resident effect of PLGA in vivo, it is necessary to analyze the DCs activation ability for with or without PLGA. As shown in Figure S13 (Supporting Information), the proportion of DCs that were activated and cross‐presenting SIINFEKL within LNs was decreased in the OVA‐DSPE hydrogel without PLGA group. This significant activation phenomenon could be attributed to enhanced antigen “hitchhiking” and LN delivery by PLGA retention.

Then, we further compared changes in peripheral multifunctional CD8^+^ IFN‐γ^+^ subsets. As shown in Figure 4J,K, the proportion of CD8^+^ IFN‐γ^+^ positive cells was also significantly increased in DSPE‐OVA‐Gel groups. These suggested that the DSPE‐OVA‐Gel vaccine could effectively improve the level of immune activation in mice. Many previous studies have shown that tumor‐infiltrating lymphocytes are key players in tumor immune surveillance.^[^ ^41^, ^42^ ^]^ Therefore, the changes of intratumoral immune subsets (CD8^+^ T, CD4^+^ T, and Treg cells) were analyzed by flow cytometry. As shown in Figure S14a,b,c andd (Supporting Information), the proportion of CD4^+^ T and CD8^+^ *T*‐cells rose notably following treatment with DSPE‐OVA‐Gel compared with the saline group. These findings were in line with our previous expectations. Additionally, DSPE‐OVA‐Gel exposure initiated the notable decrease in regulatory *T*‐cells (Treg) infiltrated in tumor tissues (from 51.0% to11.0%), demonstrating that DSPE‐OVA‐Gel treatment could also effectively remodel the tumor microenvironment and create favorable conditions for the activation of anti‐tumor immune response (Figure S14e,f, Supporting Information). Furthermore, IFN‐γ and TNF‐α are important cytokines for cytotoxic *T*‐lymphocytes to perform their effector functions and can effectively kill tumor cells.^[^ ^30^, ^43^, ^44^ ^]^ These were also obviously increased in tumor tissues (Figure 4L; Figure S15, Supporting Information) and serum (Figure 4M,N), which further confirmed the stronger antitumor immune response induced by DSPE‐OVA‐Gel. Collectively, treatment with DSPE‐OVA‐Gel enhanced the whole immune‐stimulatory effects after enhanced antigen LNs drainage, further buttressing antitumor immunity potential.

### 2.6 DSPE‐OVA‐Gel Effectively Inhibits Tumor Growth

**Figure 5:**
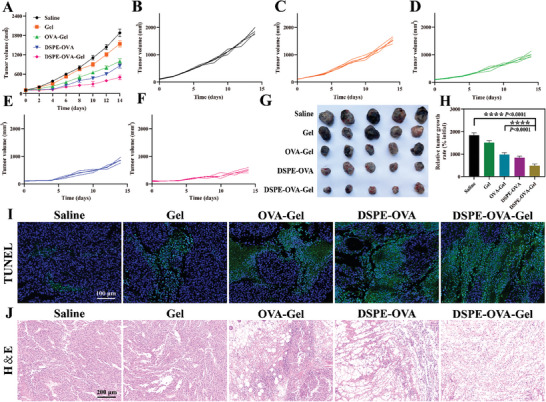
DSPE‐OVA‐Gel effectively inhibits tumor growth. A–F) Curve of tumor volume in B16‐OVA tumor bearing mice after treatment (*n* = 5 independent experiments). G) Picture of tumor tissue (*n* = 5 independent experiments). H) Relative tumor volume profiles of tumor‐bearing mice at day 21. The relative tumor volume was calculated by average tumor volume in each measured date/average tumor volume in the initial treatment date. (*n* = 5 independent experiments). I) Representative TUNEL fluorescence of mouse tumor sections (TUNEL: green; DAPI: blue, scale bar: 100 µm). J) Representative H&E staining of mouse tumor sections (scale bar: 200 µm). The results are shown as the mean ± standard deviation. Statistical analyses were performed via ordinary one‐way ANOVA with Tukey's multiple comparisons test, ^****^ *p* < 0.0001.

Next, the therapeutic effect of DSPE‐OVA‐Gel vaccines was also evaluated using the B16‐OVA tumor model. In the subcutaneous xenograft tumor model, the tumor volume was monitored, and the tumors were harvested at day 21. We found that the tumors continued to grow linearly and were macroscopically visible with a few days after saline and Gel treatment. The OVA‐Gel group and DSPE‐OVA group slowed the growth of the tumors with varying degrees (**Figure** **5**A–F). Notably, we found that DSPE‐OVA‐Gel attenuated remarkably the growth of tumors (Figure 5G,H). Subsequently, terminal deoxynucleotidyl transferase dUTP nick end labeling (TUNEL) staining and H&E staining were carried out. Mice treated with DSPE‐OVA‐Gel showed the most pronounced tumor damage and cell apoptosis (Figure 5I,J). Meanwhile, the biocompatibility and degradation of DSPE‐OVA‐Gel were investigated by implanting subcutaneously at the back of the mice. From visual observation, the hydrogel gradually degraded over time and was completely degraded after 90 days (Figure S16, Supporting Information). In addition, we found that no significant weight loss through weight monitoring (Figure S17, Supporting Information) in any group. Meanwhile, the routine blood parameters were within the normal range of fluctuations (Figure S18, Supporting Information). Therefore, the DSPE‐OVA‐Gel was biodegradable and exhibited acceptable biocompatibility for further applications.

**Figure 6:**
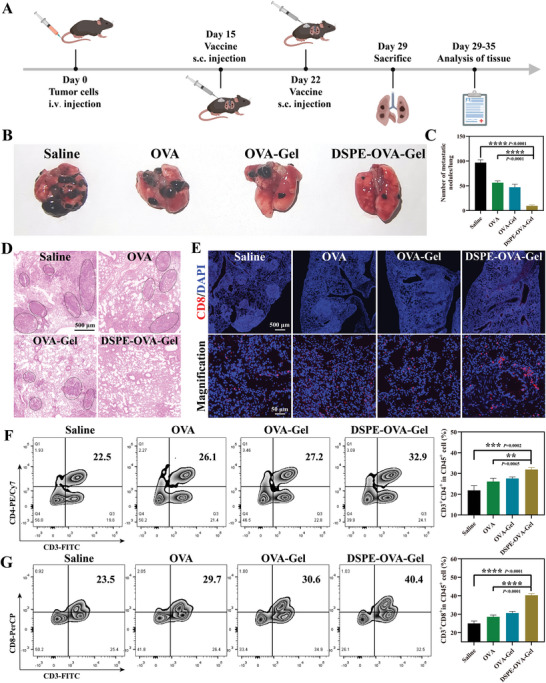
DSPE‐OVA‐Gel effectively prevents the formation of lung metastatic nodules. A) The experimental design to evaluate the efficacy of lung metastasis by the DSPE‐OVA‐Gel. Lung metastatic nodules were examined B) and counted C) at day 29 (*n* = 5 independent experiments). D) H&E staining of lungs (scale bar: 500 µm). E) Representative immuno‐fluorescence staining of CD8^+^ *T*‐cell infiltration in lung tissues (CD8: red; Nucleus: blue, scale bar of upper images, 500 µm; scale bar of bottom enlarged images, 50 µm). Representative flow cytometric analyses (left) and semi‐quantitative (right) of CD4^+^ *T*‐cells F) and CD8^+^ *T*‐cells G) (gate on CD45^+^) infiltrated in spleen after receiving different treatments in C57BL/6 mice (*n* = 3 independent experiments). Statistical analyses were performed via ordinary one‐way ANOVA with Tukey's multiple comparisons test, ^**^ *p* < 0.01; ^***^ *p* < 0.001; ^****^ *p* < 0.0001.

To further evaluate the therapeutic efficacy of the DSPE‐OVA‐Gel vaccine, we utilized the malignant melanoma with poor immunogenicity to establish another tumor syngeneic model by intravenous injection of 1 × 10^6^ B16‐F10 cells, which are known to metastasize to the lung.^[^ ^45^ ^]^ The vaccine treatments were tested using the same time course and free OVA was used as control (**Figure** **6**A). The lungs were excised for metastatic nodule quantification at day 29. As shown in Figure 6B,C, free OVA afforded only a modest protection against tumor formation, while the lung metastatic nodules in the DSPE‐OVA‐Gel group were significantly reduced. When the lungs were sectioned and stained with H&E to visualize metastases, similar findings were observed (Figure 6D). Then, we also evaluated the lung‐infiltrated CD8^+^ *T*‐cells via immuno‐fluorescence staining. As expected, the significantly higher amount of CD8^+^ *T*‐cells were detected in the lung treated with DSPE‐OVA‐Gel than the mice treated with other vaccine formulations, including OVA‐Gel (Figure 6E). Additionally, a large number of proinflammatory cytokines including IFN‐γ and TNF‐α were obviously increased at lung metastatic sites, further elucidating the immune activation effect in vivo (Figure S19, Supporting Information). Meanwhile, the infiltration and activation of immune cells in the spleen are also an important observation of the immune system, we also performed flow cytometric analyses of immune cells in the spleen and got similar results. The DSPE‐OVA‐Gel vaccine increased the proportions of CD4^+^ T (Figure 6F) and CD8^+^ T (Figure 6G) cells in the spleen, which are important components for adaptive immunity. On the whole, the DSPE‐OVA‐Gel vaccine almost equally inhibited the lung metastasis formation in the malignant melanoma with poor immunogenicity, which was consistent with the previous immune activation effect of primary tumors in vivo, indicating that the active LN accumulation system of DSPE‐OVA‐Gel could have broad applicability.

## 3 Discussion

This study focuses on the frontier hotspots and urgent needs of tumor immunotherapy. Although therapeutic cancer vaccines have made significant breakthroughs in clinical trials of some malignant tumors, the clinical efficacy of the vaccines still needs improvement.^[^ ^15^ ^]^ Lymph nodes are the primary organs that initiate adaptive immune responses, however, the low targeted delivery efficiency of the vaccine limits the immune efficacy.^[^ ^12^ ^]^ Therefore, the development of innovative safe, and effective lymph node‐targeted vaccine‐delivery systems are imperative to improve the clinical efficacy of antigen vaccines. Current LN‐targeting strategies have been centered around improving the transfer efficiency from peripheral tissues to lymphatic vessels by changing the physicochemical properties (for example, size, charge, and deformability) of nano‐vaccines, but the inherent limitations of passive transport in nano‐vaccines have long been neglected.^[^ ^46^, ^47^ ^]^ To address the above‐mentioned bottlenecks, the present study has taken an alternative approach to design a vaccine active delivery system with specific LN‐targeting by “hitchhiking”.

Inspired by the Migrated‐DCs homing from the periphery to the LNs, we successfully developed an injectable hydrogel vaccine, which induced potent antigen depot at the administration sites to recruit and “hitchhike” with Migrated‐DCs for the active targeting and accumulation of antigens in LNs. Through a comprehensive assessment, including fluorescence intensity measurements of the LN sections, in vitro imaging, and flow cytometry to determine the proportions of LN‐resident or migrated DCs, the enhanced LN drainage exhibited by the hydrogel antigen depot in subcutaneous tissue indicated that recruiting and “hitchhiking” subcutaneous DCs might offer an alternative strategy for the potent LN transfer of vaccines. Additionally, LNs are the main places where immune responses occur, and harbor more abundant APCs compared to subcutaneous tissues.^[^ ^19^ ^]^ The antigen sharing by Migrated‐DCs with a large number of LN‐resident APCs is achieved through releasing from the Migrated‐DCs under the acidic microenvironment within the LNs using pH‐sensitive bonds between antigen and DSPE. Based on this process, an “One‐to‐Many” (One Migrated‐DC corresponding to Many LN‐resident APCs) strategy, potent antigen‐specific immune responses could be induced. In melanoma tumor model, the in‐situ hydrogel could greatly promote the DCs maturation, induce sufficient T lymphocyte in LNs, eventually increase the proportion of CD8^+^ and CD4^+^ T cells in tumors, and effectively control the growth of tumor and lung metastasis. These findings provide a feasible strategy for enhancing the efficacy of clinical cancer vaccines.

In addition, adaptive immune responses are primarily initiated in secondary lymphoid organs. Accordingly, LNs are attractive therapeutic targets for the treatment of a variety of unmet clinical needs. DCs, as widely present immune cells in the body, can enhance the delivery dose to LNs and trigger potent adaptive immune signaling through “hitchhiking” with endogenous DCs only by DSPE modification of different antigens, which is expected to achieve early prevention and treatment of a variety of diseases. Importantly, the hydrogel is expected to further extend the durability of immune activation in clinical vaccines by combining the characteristics of a high drug loading rate and improved antigen stability in vivo. However, optimizing the process of the preparation and ensuring preservation for transport is critical for clinical translation. Therefore, we consider optimizing the hydrogel preparation process to increase the clinical applicability.

## 4 Experimental Section

### Materials

OVA (SIINFEKL), FITC‐OVA and 5‐TAMRA‐OVA were purchased from BankPeptide (Hefei, China). DSPE‐PEG‐benzaldehyde (DSPE) was purchased from Ruixibio (Xian, China). Sodium alginate was purchased from Solarbio Biotechnology. Polylactic acid hydroxy acetic acid copolymer (molecular weight [MW] = 37–52 kDa; lactic acid: glycolic acid = 75:25) was purchased from Yuanye Biotechnology (Shanghai, China). PE anti‐Foxp3 antibody was purchased from Miltenyi Biotec GmbH. APC anti‐CD45 antibody, FITC anti‐CD3 antibody, PE/Cy7 anti‐CD4 antibody, PerCP anti‐CD8 antibody, Brilliant Violet 421 anti‐CD11c antibody, PE anti‐CD11c antibody, Brilliant Violet 650 anti‐CD86 antibody, PE/Cy5 anti‐CD86 antibody, APC anti‐H2Kb/SIINFEKL antibody and APC anti‐CCR7 antibody were purchased from Biolegend (San Diego, CA). Other reagents and solvents were purchased from Thermo Fisher Scientific and used as received unless otherwise stated.

### Cell Line and Cell Culture

B16‐F10 mouse melanoma cells expressing OVA protein (B16‐OVA) and dendritic cell line DC2.4 were purchased from the China Typical Culture Collection (Wuhan, China). B16‐F10 mouse melanoma cells and RAW 264.7 mouse macrophages cells obtained from iCell Bioscience Inc. (Shanghai, China). These cells were all cultured in RPMI 1640 medium supplemented with 10% FBS and 1% penicillin‐streptomycin solution at 37 °C under 5% carbon dioxide.

### Animals

The female C57BL/6 mice (SPF class) were purchased from the Spelford Laboratory Animal Centre (Beijing, China). All Animals were fed in the condition of 25 °C and 55% relative humidity in the Experimental Animal Center of Zhengzhou University. All mice were maintained under specific pathogen‐free conditions with a 12 h light/12 h dark cycle. All animal handling procedures were approved by the guidelines of the Institutional Animal Care and Use Committee of Zhengzhou University (yxy11sc20220099).

### Synthesis of DSPE‐OVA

OVA (10 mg, 1.0 eq), DSPE (36 mg, 1.2 eq), and DMAP (2.54 mg, 2.0 eq) were dissolved in 2 mL of DMSO in anhydrous conditions. Then, it was sonicated and transferred to a 10 mL round‐bottom flask with a pipette to mix. The reaction was stirred magnetically at room temperature for 72 h. After the reaction was completed, the reaction solution was transferred to dialysis bags (MWCO: 2000) and dialyzed for 48 h with 0.01 m NaHCO_3_ solution (pH 8.0–8.5) as the medium, during which the dialysate was changed every 6 h. Finally, the dialysis samples were freeze–dried to obtain a white powder and stored at −20 °C. For the preparation of FITC and 5‐TAMRA labeled DSPE‐OVA, the OVA was replaced with the corresponding fluorescently labeled peptide during the synthesis process, and the rest of the experimental conditions were unchanged. The products were characterized by FT‐IR and ^1^H‐NMR.

### Synthesis of PLGA Porous Microspheres

PLGA porous microspheres were prepared using a porogenic agent combined with the compound emulsion volatilization (W/O/W) method. The steps were as follows: 150 mg PLGA was prepared as organic phase O by dissolving in 3 mL dichloromethane (DCM), and 60 mg BSA was dissolved in 0.8 mL deionized water as aqueous phase W_1_ using bovine serum albumin BSA as the porogenic agent. The two solutions were mixed and sonicated in an ice bath (60 W, 2 min, working for 3 s, stopping for 5 s) to emulsify them to form W_1_/O colloid; the resulting colloid was transferred to 300 mL of 0.4% PVA aqueous solution to obtain a W_1_/O/W_2_ double emulsion, which was mechanically stirred at 900 rpm min^−1^ for 4 h at room temperature. After the organic solvent evaporation and the microspheres hardening, the microspheres were collected and washed three times with deionized water. Finally, the microspheres were freeze–dried to obtain a white powder and stored at ‐20 °C. To characterize the microspheres, the lyophilized microspheres were adhered to a conductive gel and the surface of the microspheres was gold sprayed and scanned using SEM at a voltage of 3 kV.

### Preparation of DSPE‐OVA‐Gel Vaccine

The 200 mg of sodium alginate was dissolved in 10 mL of deionized water and stirred magnetically for 4 h at 45 °C to dissolve. After cooling to room temperature, DSPE‐OVA (40 mg mL^−1^) PLGA porous microspheres (4%) and GM‐CSF (10 µg mL^−1^) were added to sodium alginate solution, and stirred well. In vitro, the mixed hydrogel solution was slowly injected into the solution with 1.8 × 10^−3^ mol L^−1^ CaCl_2_ and 1.5 × 10^−3^ mol L^−1^ MgCl_2_ using a syringe. The mixture was then allowed to stand at room temperature for 2 h until gel formation. In vivo, subcutaneous injection of DSPE‐OVA‐Gel was able to form a hydrogel directly. SEM was used to observe the morphology of DSPE‐OVA‐Gel at an accelerating voltage of 3 kV. The hydrogel was lyophilized and coated with a thin layer of gold prior to observation.

### GM‐CSF and DSPE‐OVA Release from the DSPE‐OVA‐Gel In Vitro

The DSPE‐OVA‐Gel was placed in the PBS at 37 °C, and the solution was taken out at the required time points. The content of GM‐CSF was measured with an ELISA kit and FITC‐labelled DSPE‐OVA was used to detect the OVA release. Finally, the concentration was calculated by detecting the fluorescence intensity at different time points. The cumulative release percentage of GM‐CSF and OVA in the DSPE‐OVA‐Gel was calculated, and the in vitro release curve was drawn.

### The Recruitment of DCs by DSPE‐OVA‐Gel

A transwell migration assay was implemented to study the recruitment of DCs. Transwell plates with 8 µm pore filters on the bottom of the upper compartment were used to study DCs migration. DSPE‐OVA‐Gel encapsulating GM‐CSF (10 µg mL^−1^), or without GM‐CSF were placed in the lower compartment of the transwell, while 5 × 10^4^ DCs labeled with 0.25 µmol L^−1^ CFSE were seeded in the upper compartment. After incubation for 24 h, cells migrated into the lower compartment were visualized using CLSM (Leica TCS SP8, Germany), the DCs in the hydrogel were then collected and counted.

To demonstrate the in vivo recruitment of DCs, 100 µL of DSPE‐OVA‐Gel with or without GM‐CSF was injected subcutaneously into the right dorsum of C57BL/6 mice, three in each group. The mice were dissected at day 5, 10, and 15. The hydrogel with surrounding tissues was carefully peeled off with dissecting scissors, rinsed with PBS, and placed in 1.5 mL EP tubes. The large pieces of hydrogel were cut up with scissors. After lysis by alginate lyase, the supernatant was discarded by centrifugation ( rpm, 10 min), and the precipitate was washed twice with PBS. Then, 500 µL of PBS was added to resuspend the cell precipitate, and the total number of cells was counted using flow‐through absolute microspheres. Specifically, 100 µL of cell suspension was slowly added to the flow counting tube using a pipette, taking care to avoid contact between the tip of the gun and the liquid surface, then the counting tube was flicked to allow the cell suspension to fully bind to the microspheres. Finally, the cells were sampled and detected by flow cytometry (BD LSR Fortessa).

Meanwhile, the corresponding amount of Brilliant Violet 421‐CD11c antibody was added to the cell suspension according to the flow antibody instructions, and the cells were incubated on ice for 30 min in the dark, during which the cells were lightly stirred every 10 min to allow the antibody to fully bind to the cells. Subsequently, the cell suspension was centrifuged (2000 rpm, 10 min). Finally, the precipitate was washed once with PBS and resuspended in 500 µL PBS for flow cytometry analyses. The number of DCs within the hydrogel was calculated based on the total number of cells and the proportion of DCs obtained by flow cytometry analyses.

For a more visual observation of the recruitment of cells in the hydrogel, the subcutaneous hydrogel was carefully peeled from the back of the mice at day 15, fixed using 4% paraformaldehyde fixative, paraffin‐embedded and dehydrated, followed by H&E staining.

### Analyses of DCs Retention in DSPE‐OVA‐Gel

To investigate whether PLGA porous microspheres could promote cell retention in hydrogels, the number of cells that migrated to the lower chamber in the same period by transwell experiments were examined. The prepared gel with/without porous microspheres (4%) and 5×10^4^ DCs were placed in the upper chamber of the transwell, while 650 µL of complete medium containing 15% FBS was added to the lower chamber and incubated in a cell culture chamber. After 24 h of migration, the cells in the lower chambers of the transwell were photographed using CLSM.

### Analysis of the Anchoring Ability of DSPE‐OVA on DCs

First, it was examined that whether DSPE‐OVA could be anchored to the cell membrane of DCs in solution. FITC‐labelled DSPE‐OVA was used in this experiment. DCs were seeded in confocal dishes at a concentration of 3 × 10^5^ cells/dish. After 12 h, the cells were incubated for 1 h with the amiloride (AMI, 100 µg mL^−1^) to inhibit cellular uptake. Next, DSPE‐OVA‐containing medium (400 µg mL^−1^) was added to incubate DCs for 2 h at 37 °C in the dark. The cell membrane red fluorescent probe 1,1′‐dioctadecyl‐3,3,3′,3′‐tetramethylindocarbocyanine perchlorate (DiI, 5 µm) was used to stain cell membranes for 10 min and washed 3 times with PBS. Then,1 mL of PBS was added for observation and image by CLSM.

Next, 100 µL of gel coated with DSPE‐OVA (FITC) was prepared. DCs were diluted to 5 × 10^5^ cells mL^−1^ using serum‐free medium. Pre‐prepared hydrogels were placed in the upper chamber and 100 µL of cell suspension was added to the upper layer of the gel. Then, the lower chamber was placed in a 24‐well plate round cell crawl and 650 µL of complete medium containing 15% FBS was added, placed in a cell culture incubator. After 24 h of migration, the cell in lower chamber were stained with DiI for 10 min and washed 3 times with PBS. Finally, add a drop of antifade mounting medium to the slide, place the cell upside down on the slide, and finally observe by CLSM.

### Analyses of the Migration Ability of DCs Loaded with DSPE‐OVA

DCs were spread in a six‐well plate at a concentration of 3 × 10^5^ cells/well, 1 mL of complete medium containing DSPE‐OVA (400 µg mL^−1^) was added, and blank medium was used as control. 200 µL of cell suspension was added to the upper chamber of the transwell 24‐well plate and 650 µL of complete medium containing 15% FBS was added to the lower chamber. Then, it was placed in a cell culture incubator at 37 °C in the dark. After migrating for the other 24 h, cells were fixed and stained with 1% crystal violet, cells on the upper side of each insert were gently removed with a cotton swab. Migrated cells were imaged and counted by microscopy. Unmodified DCs in the 24‐well plate were served as a positive control.

### Analyses of DCs Maturation and Migration in DSPE‐OVA‐Gel

DC2.4 (5 × 10^4^/well) and hydrogel vaccine with different components were transferred to the upper chamber of the transwell, and CCL21 (1 µg mL^−1^) was added to the lower chamber. After 24 h of treatment, cells in the lower chamber and hydrogel were harvested. Flow cytometry was performed to detect the proportion of CD86^+^ and CCR7^+^ cells.

To investigate the relative contribution of CCR7 to the migration of DCs toward CCL21, the collected DC2.4 cells were treated with or without anti‐CCR7‐neutralizing antibody (20 µg mL^−1^) for 30 min. Migration assays were performed by using 8 µm pore cell culture inserts. 200 µL of collected cell suspension was added to the upper chamber of the transwell 24‐well plate and 650 µL of complete medium containing 1 µg mL^−1^ CCL21 was added to the lower chamber and placed in a cell culture incubator at 37 °C. After migrating for th other 24 h, cells were fixed and stained with 1% crystal violet, cells on the upper side of each insert were gently removed with a cotton swab. Migrated cells were imaged and counted by microscopy.

### Analyses of OVA Acid‐Reactive Release and Reuptake by Resident DC Cells

OVA acid‐reactive release: The acid‐stable fluorescein 5‐TAMRA was used to label the N‐terminal end of OVA to synthesize DSPE‐OVA in this experiment. DCs were seeded in confocal dishes at a concentration of 3 × 10^5^ cells/dish. After 12 h, the original medium was discarded, and 1 mL of complete medium containing DSPE‐OVA (5‐TAMRA) (400 µg mL^−1^) was added and incubated at 37 °C for 2 h in the dark. Then incubated at pH 7.4 and pH 6.4 for 1 h, respectively, and finally observed by CLSM.

The uptake behavior of reuptake by resident DCs in vitro: DCs were seeded at six‐well plates at a concentration of 3 × 10^5^ cells/well. Then, 1 mL of complete medium containing DSPE‐OVA (5‐TAMRA) (400 µg mL^−1^) was added and incubated at 37 °C for 2 h in the dark. Finally, DCs treated with a complete medium at pH 6.4 for 1 h. The cell culture medium was collected and the total amount of release from the supernatant was calculated by OVA calibration curve. Meantime, the cell culture medium was added to a blank DCs culture dish for 6 h. The cells were collected by digestion and finally measured by flow cytometry.

### In Vivo Accumulation of DSPE‐OVA‐Gel in LNs

Female C57BL/6 mice (6–8 weeks old) were injected subcutaneously with DSPE‐OVA‐Gel, OVA‐Gel, and free OVA (100 µL, *n* = 3 per group, OVA levels were consistent in each group). Inguinal and axillary LNs adjacent to the injection site were harvested from mice at 0, 12, 24, 48, and 72 h after injection, and imaged using a small animal imaging system (Xtreme, Bruke). The LNs were then isolated at 24 h and cryo‐sectioned to a thickness of 8 µm, followed by immune‐fluorescence staining to image the location of OVA in the LNs. Next, the systemic distribution of DSPE‐OVA‐Gel injected subcutaneously into 6–8‐week‐old female C57BL/6 mice was investigated. LNs and other organs were harvested from the mice after 0, 12, 24, 48, and 72 h after injection and imaged ex vivo by a small animal imaging system.

To distinguish Migrated‐DCs and LN‐resident DCs uptake of OVA in LNs. After injection 72 h, LNs were excised and pushed through a 70 µm cell strainer to generate a single cell suspension. The cells were further stained with CD11c and CD11b monoclonal antibodies to distinguish Migrated‐DCs and LN‐resident DCs in the LNs by flow cytometry.

### Immune Response Induced by the DSPE‐OVA‐Gel System

Female C57BL/6 mice of 6–8 weeks were used to construct animal models. 2 ×10^6^ B16‐OVA cells in 100 µL PBS were subcutaneously injected on the right wing of each mouse. After 6 days later, the tumor volume of the mice reached about 100 mm^3^. Then, the mice were randomly divided into five groups of different treatments (*n* = 5): Saline, Gel, OVA‐Gel, DSPE‐OVA, and DSPE‐OVA‐Gel. To ensure consistent vaccination doses, 100 µL vaccine (OVA levels were consistent in each group) was given to each mouse of different compositions. All mice were euthanized at day 21, and inguinal LNs adjacent to the injection site were removed and photographed. The draining LNs were collected to analyze the proportion of CD11c^+^ CD86^+^, CD11c^+^ MHC‐II^+^, CD4^+^ and CD8^+^ T cells. Then, an anti‐CD11c and anti‐SIINFEKL/H‐2Kb were used to detect the specific H‐2Kb–restricted peptide on DCs surface within LNs. Meanwhile, IFN‐γ^+^ CD8^+^ *T*‐cells in the peripheral blood were analyzed. The serum was separated, and the secretion of proinflammatory cytokines TNF‐α and IFN‐γ was detected by ELISA. The tumor tissue was carefully peeled off using a dissecting tool, and dissociated into a single‐cell suspension according to the tumor tissue single‐cell lysis kit instructions. The cell suspension was stained with anti‐CD45‐APC, anti‐CD3‐FITC, anti‐CD4‐PE/Cy7, anti‐CD8‐PerCP for *T*‐cell detection, and anti‐CD45‐APC, anti‐CD3‐FITC, anti‐CD4‐PE/Cy7, anti‐FoxP3‐PE for Treg cell detection. Flow cytometry acquisition was performed on a flow cytometer and data analyses was performed with FlowJo (Version 10).

### Immuno‐Fluorescence Analyses of Cytokine Secretion in Tumor Tissues

The tumor was placed in 4% paraformaldehyde for 24 h at 4 °C, then transferred to 15% and 30% sucrose solution (w/w) for dehydration. The tumor was implanted in the optimal cutting medium and the frozen section was placed in a cryostat microtome. The tissue sections were rinsed with PBS, permeated, and then sealed at room temperature with 5% bovine serum albumin (BSA) for 1 h. Finally, the tissue sections were stained with different major antibodies: IFN‐γ, TNF‐α, and DAPI overnight at 4 °C, as per manufacturer's instructions. After adding fluorescently labeled secondary antibodies, the slides were analyzed by confocal microscopy.

### In Vivo Antitumor Effect of the DSPE‐OVA‐Gel System and Safety Analyses

After vaccination of tumor‐bearing mice, the tumor volume and body weight of the mice were recorded every two days for 14 days. The tumor volume was measured via the following equation: V = (width^2^ × length)/2. All mice were euthanized at day 21, blood samples were collected for blood routine analyses, and the tumors were taken out and photographed. The tumors were fixed immediately in 4% paraformaldehyde, followed by standard dehydration and paraffin embedding. The embedded tissues were then sectioned into 4 µm slices and then subjected to standard H&E and TUNEL staining for histological analyses.

For the assessment of lung metastasis inhibition by DSPE‐OVA‐Gel, B16‐F10 melanoma cells (1 × 10^6^ cells per mouse) were injected intravenously into C57BL/6 female mice (*n* = 5 per group). 14 days later, the mice were injected subcutaneously with saline, free OVA, OVA‐Gel, and DSPE‐OVA‐Gel. At day 29, the animals were euthanized, lungs were removed and photographed. The number of metastatic nodules was counted. Next, the lungs were processed for H&E staining, immuno‐fluorescence staining for infiltrating CD8^+^ *T*‐cells, and cytokine secretion (IFN‐γ and TNF‐α). In order to detect the number of *T*‐cells in the spleen of mice, splenocytes were collected, and flow cytometry was performed to detect the proportion of CD4^+^ /CD8^+^ *T*‐cells in the spleen, and data analyses were performed with FlowJo (Version 10).

### Statistical Analysis

Unless otherwise stated, all experiments were replicated independently at least three times. Data are presented as mean ± SD. Statistical analyses statistics were performed using GraphPad Prism 8.0. Statistical analyses were performed via unpaired two‐tailed Student's *t*‐tests and one‐way ANOVA with Tukey's correction. For all tests, ns. meant not significant: *p* > 0.05, ^*^ *p *< 0.05, ^**^ *p *< 0.01, ^***^ *p *< 0.001 and ^****^ *p *< 0.0001.

## Conflict of Interest

The authors declare no conflict of interest.

## Author Contributions

J.J.L., J.J.S., and L.Z. conceived and coordinated the study. Z.Z.Z. supervised the project. L.Z., L.Z., P.L.S., and M.Y.W. performed the experiments. X.Q., Q.Y.S., and X.Z. participated in performing experiments and discussing the results; D.Y.X., and M.H.M. analyzed the data and wrote the paper. All authors discussed the results and have given approval to the final version of the paper.

## Supporting information

Supporting Information

## Data Availability

The data that support the findings of this study are available from the corresponding author upon reasonable request.
