# Supplementary material for: Dendritic Cell‐Hitchhiking In Vivo for Vaccine Delivery to Lymph Nodes
Source: Adv Sci (Weinh). 2024 Jul 4;11(33):2402199. doi: 10.1002/advs.202402199 (PMC11434131; doi:10.1002/advs.202402199)
Supplement: Supplementary file 1 — Supporting Information [file ADVS-11-2402199-s001.docx]

Supporting Information

**Dendritic Cell-Hitchhiking *in Vivo* for Vaccine Delivery to Lymph Nodes**

*Lei Zhou, Ling Zhao, Mengyao Wang, Xu Qi, Xin Zhang, Qingying Song, Dayu Xue, Meihua Mao, Zhenzhong Zhang*, Jinjin Shi *,* *Pilei Si *, Junjie Liu **

Lei Zhou, Ling Zhao, Mengyao Wang, Xu Qi, Xin Zhang, Qingying Song, Dayu Xue, Meihua Mao, Prof. Zhenzhong Zhang, Prof. Jinjin Shi, Prof. Junjie Liu

School of Pharmaceutical Sciences, Zhengzhou University, Zhengzhou 450001, China.

Email: liujunjie@zzu.edu.cn (J.L.). shijinyxy@zzu.edu.cn (J.S.). zhangzhenzhong@zzu.edu.cn (Z.Z.).

Prof. Pilei Si

Department of Breast Surgery, Henan Provincial People’s Hospital, People’s Hospital of Zhengzhou University, People’s Hospital of Henan University, Zhengzhou, 450003, Henan, China

Email: [siplei2013@pku.edu.cn](mailto:siplei2013@pku.edu.cn)

Prof. Zhenzhong Zhang, Prof. Jinjin Shi, Prof. Junjie Liu

Key Laboratory of Targeting Therapy and Diagnosis for Critical Diseases, Zhengzhou 450001, China.

**This PDF file includes:**

Figs. S1 to S19


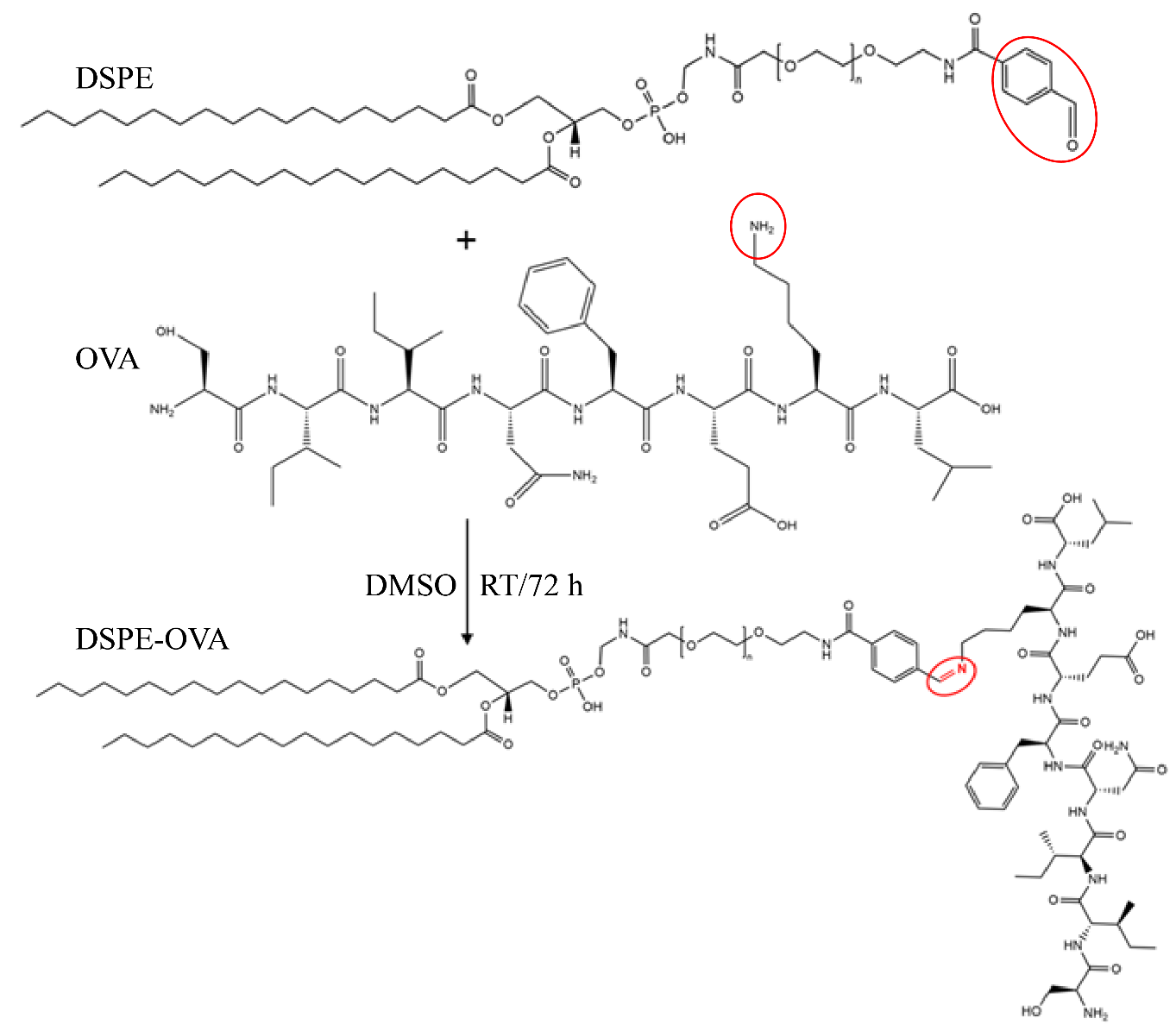


**Figure S1.** The synthetic route of DSPE-OVA.


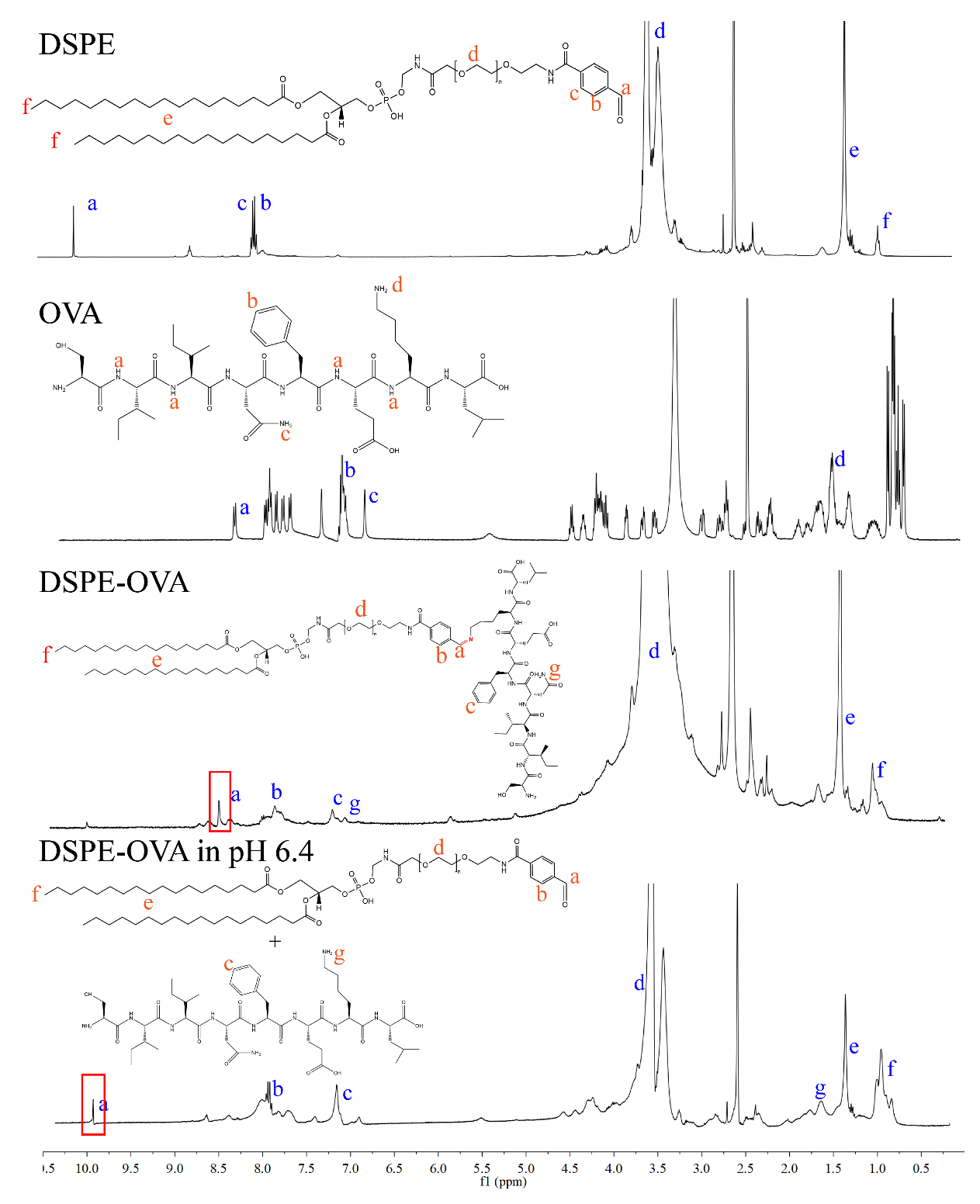


**Figure S2.** ^1^H-NMR spectra of DSPE, OVA, DSPE-OVA in pH 7.4 and DSPE-OVA in pH 6.4. The spectra displayed signals at 10.1 ppm corresponded to the aldehyde protons of the DSPE, and the peaks of OVA at 1.5 ppm corresponded to the primary amine protons on the side chain of lysine. After formation of the copolymer DSPE-OVA, the aldehyde peak of DSPE (10.1 ppm) and the primary amine protons of OVA (1.5 ppm) disappeared, while the imine protons at 8.5 ppm were seen. These results indicated that DSPE-OVA was successfully synthesized. Subsequently, the DSPE-OVA was pre-treated with aqueous solution of pH 6.4 for 12 h. The aldehyde proton peak (10.1 ppm) appeared and the imine proton peak (8.5 ppm) was not detected, illustrating the hydrolysis of DSPE-OVA.


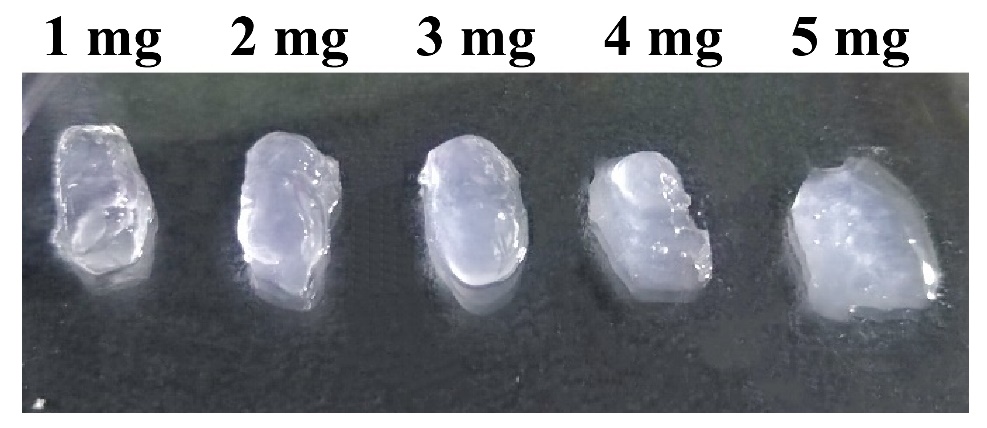


**Figure S3.** Investigation of the maximum DSPE-OVA loading in DSPE-OVA-Gel. The results showed that the hydrogel still had a good solid structure when the DSPE-OVA concentration was 4 mg/100 μL, while the hydrogel was more fluid when the mass increased to 5 mg/100 μL.


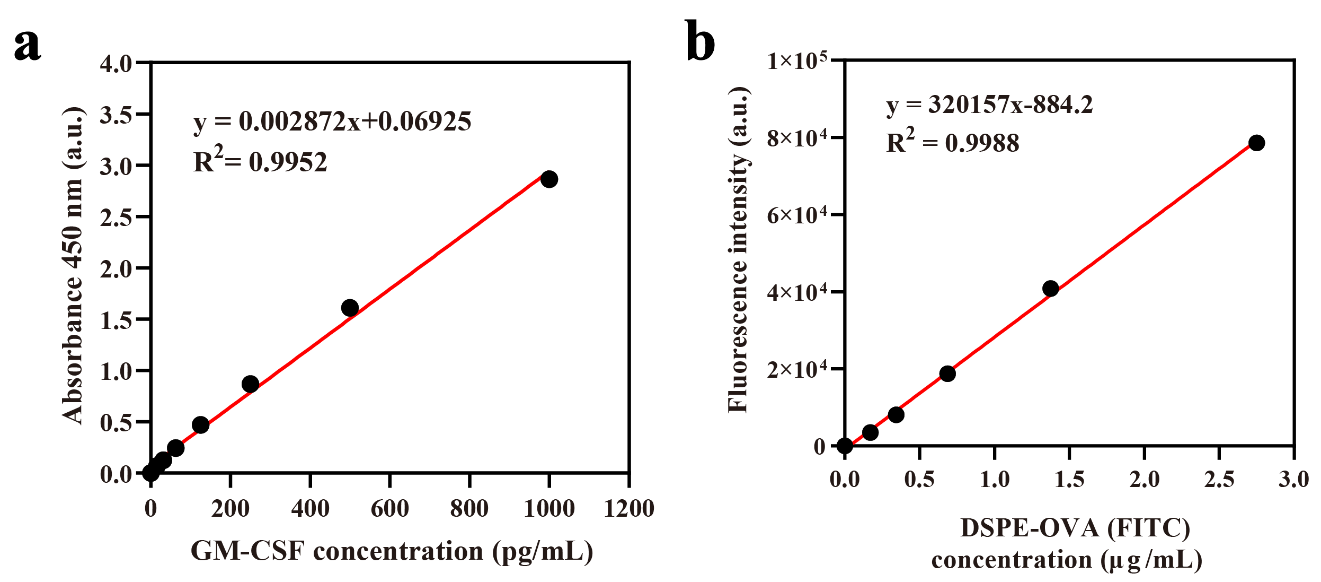


**Figure S4.** (a) The standard curve and linear relationship for GM-CSF concentrations. (b) The standard curve and linear relationship for fluorescence intensity versus the concentrations of DSPE-OVA (FITC).


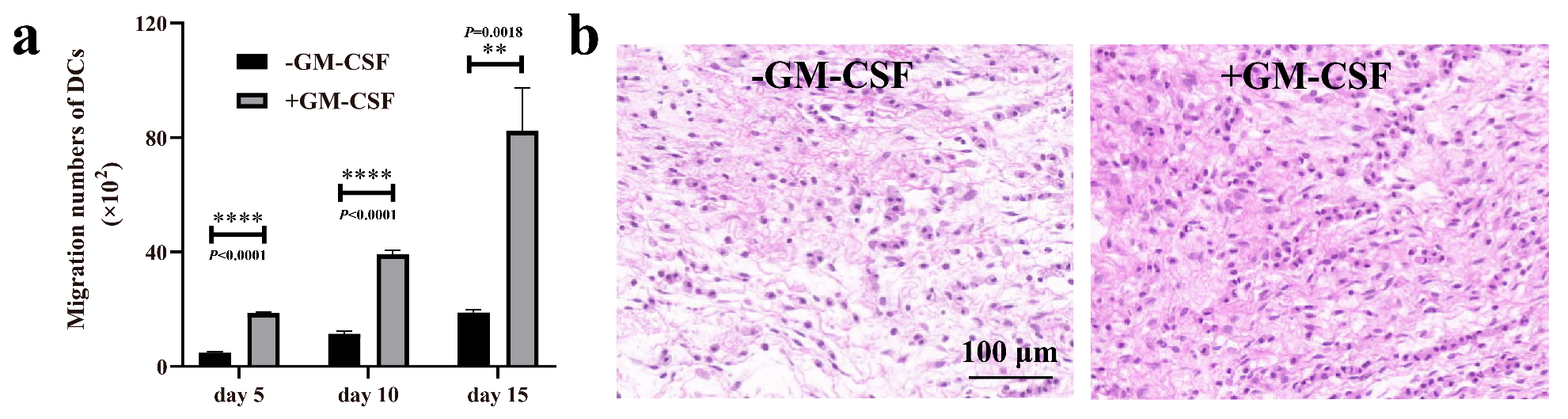


**Figure S5.** (a) The DCs recruitment ability of hydrogel at 5, 10 and 15 days after subcutaneous injection. (b)H&E staining of sectioned hydrogel on 15th day after subcutaneous injection (scale bar: 100 μm). The results are shown as the mean ± standard deviation. Statistical analyses were performed via the two-sided Student’s t-test, ***p* < 0.01; *****p* < 0.0001.


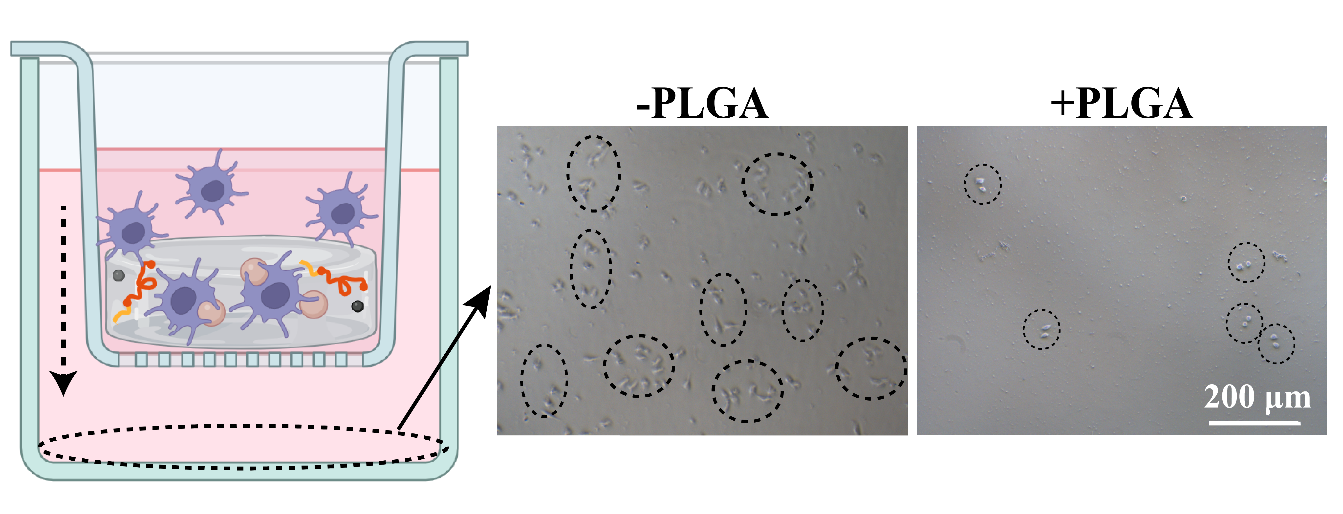


**Figure S6.** Transwell migration assay investigated the time prolongation of DCs residence in the hydrogel by porous PLGA microspheres (scale bar: 200 μm).


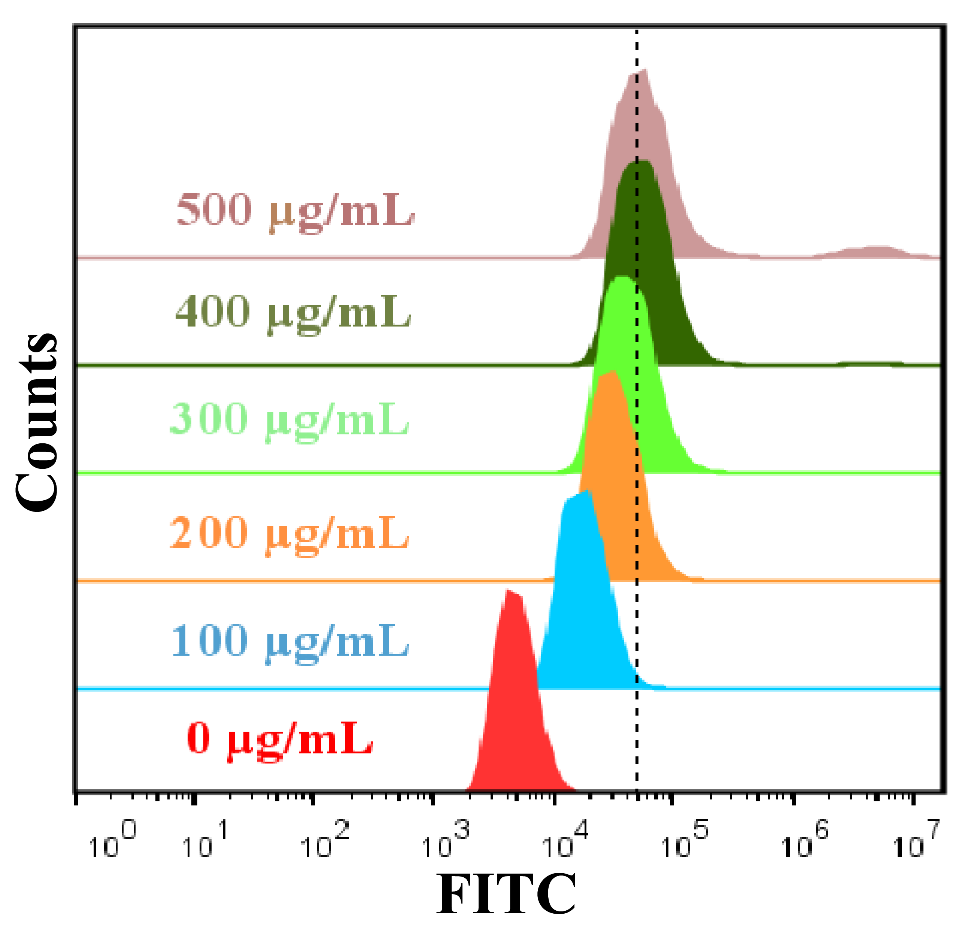


**Figure S7.** Flow analyses results of maximum DSPE-OVA concentration to load on DCs.


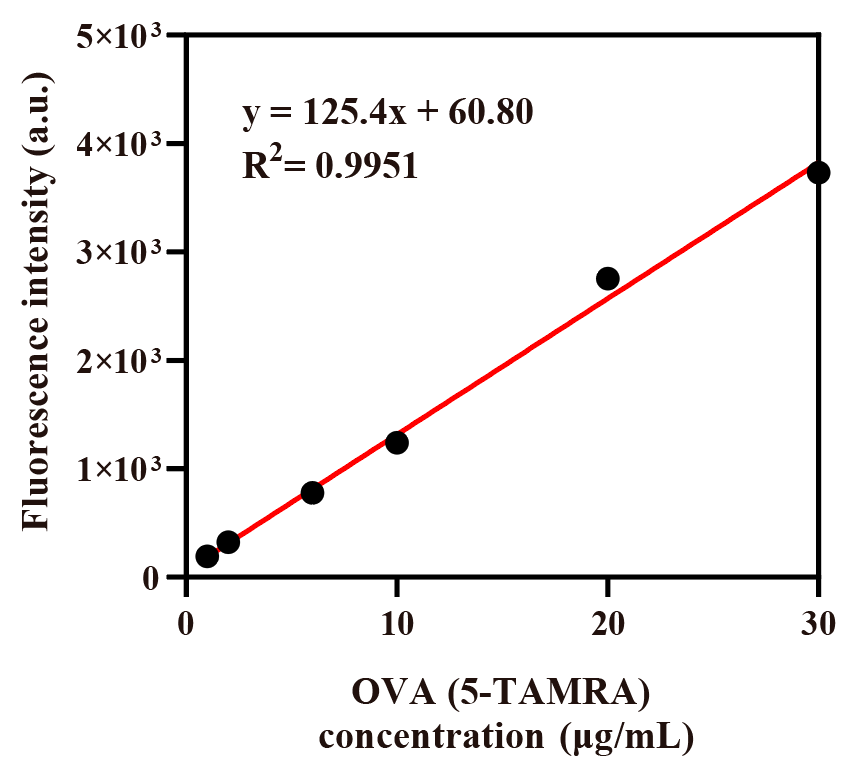


**Figure S8.** The concentration–fluorescence intensity standard curve of TAMRA-labeled OVA.


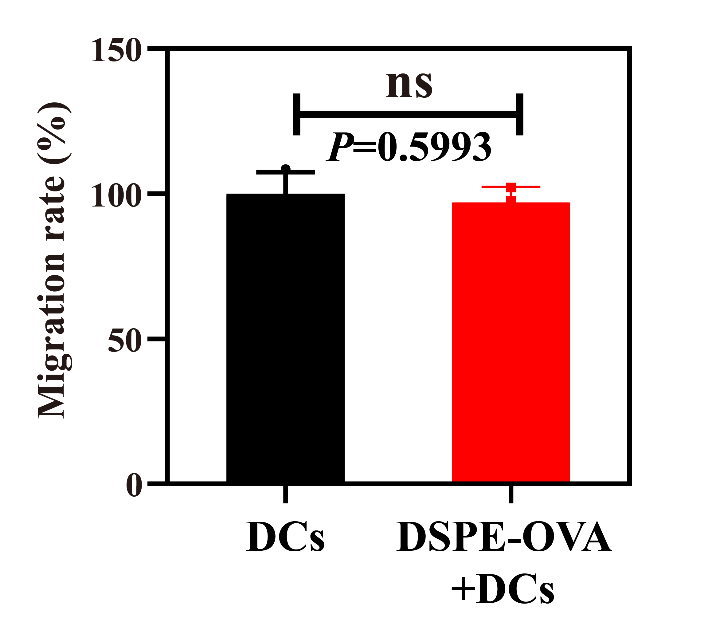


**Figure S9.** The migration ability of DCs loaded with DSPE-OVA was evaluated by transwell coculture system (n =3 independent experiments). The results are shown as the mean ± standard deviation. Statistical analyses were performed via the two-sided Student’s t-test, no significant (ns): *p* > 0.05.


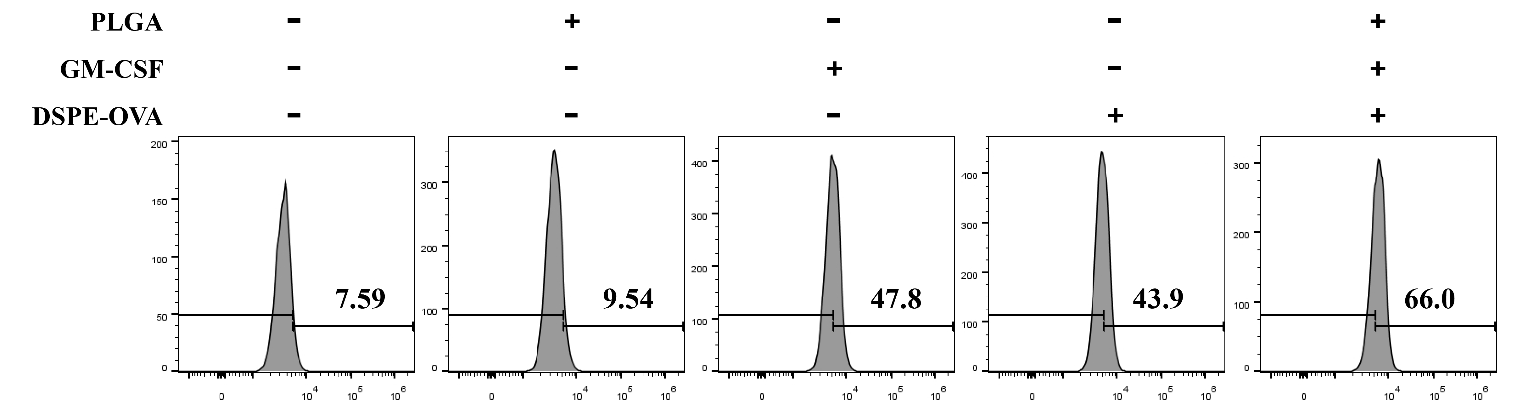


**Figure S10.** Flow cytometry for surface expression of CD86 in DC2.4 cells after the hydrogel treatment.


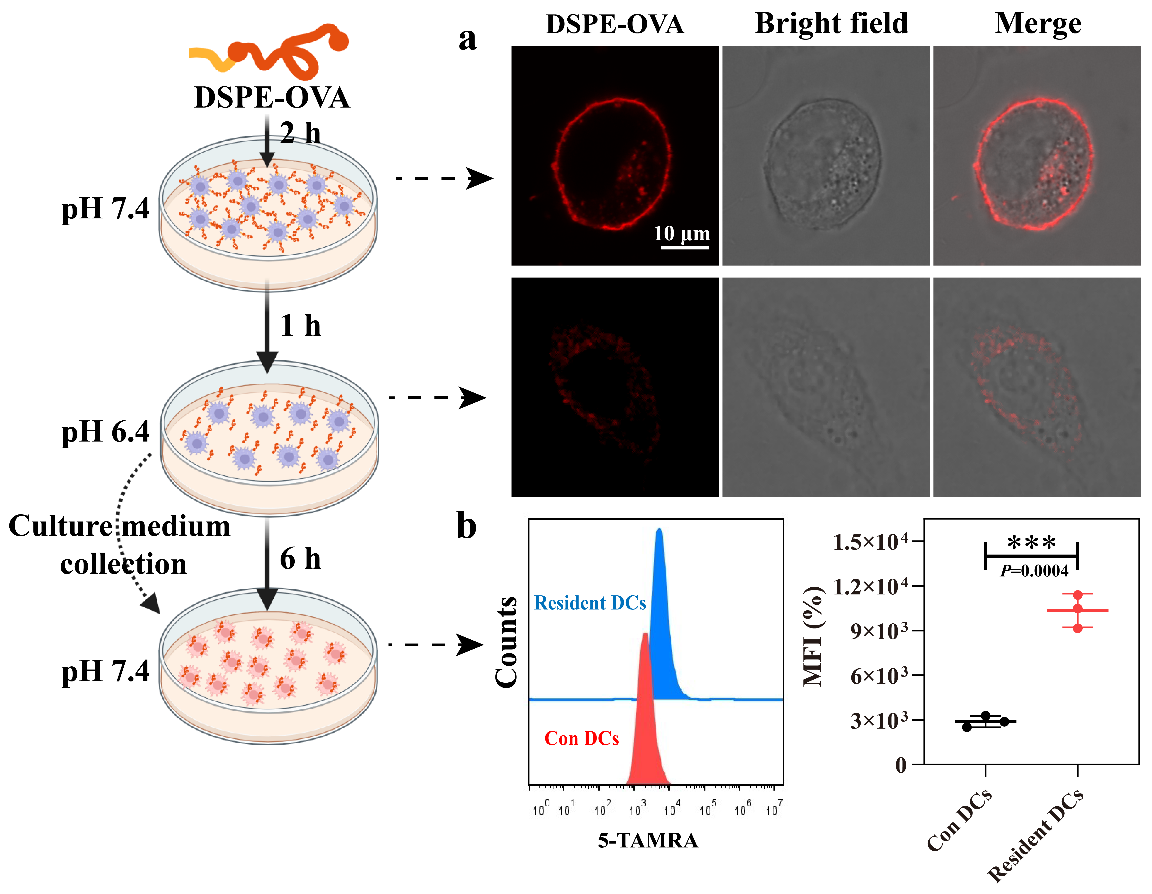


**Figure S11.** (a) Representative CLSM images of OVA acid-response release (scale bar: 10 μm). (b) Representative flow cytometric analyses (left) and relative quantification (right) of reuptake behavior by Resident-DCs (n = 3 independent experiments). The results are shown as the mean ± standard deviation. Statistical analyses were performed via the two-sided Student’s t-test, ****p* < 0.001.


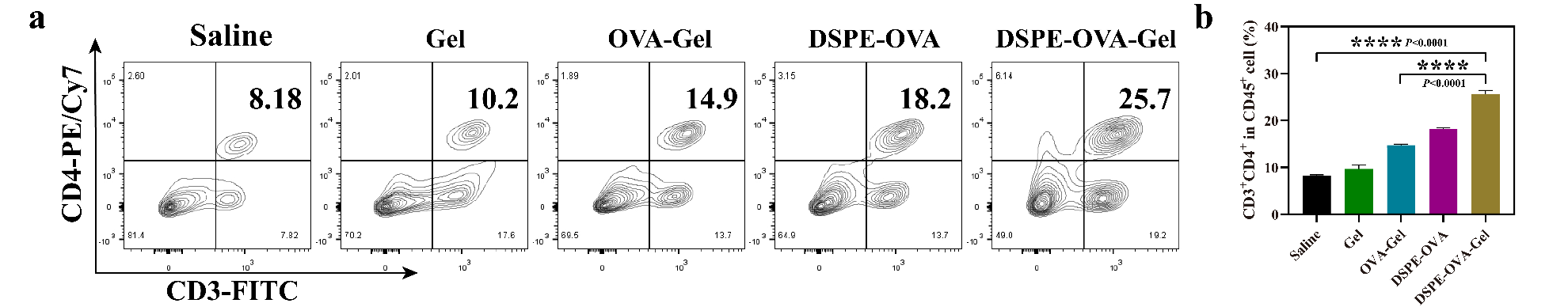


**Figure S12.** Representative flow cytometric analyses (a) and semi-quantitative (b) of CD3^+^ CD4^+^ T cells (gate on CD45^+^) infiltrated in LNs after receiving different treatments in C57BL/6 mice (n = 3 independent experiments). The results are shown as the mean ± standard deviation. Statistical analyses were performed via ordinary one-way ANOVA with Tukey’s multiple comparisons test, *****p* < 0.0001.


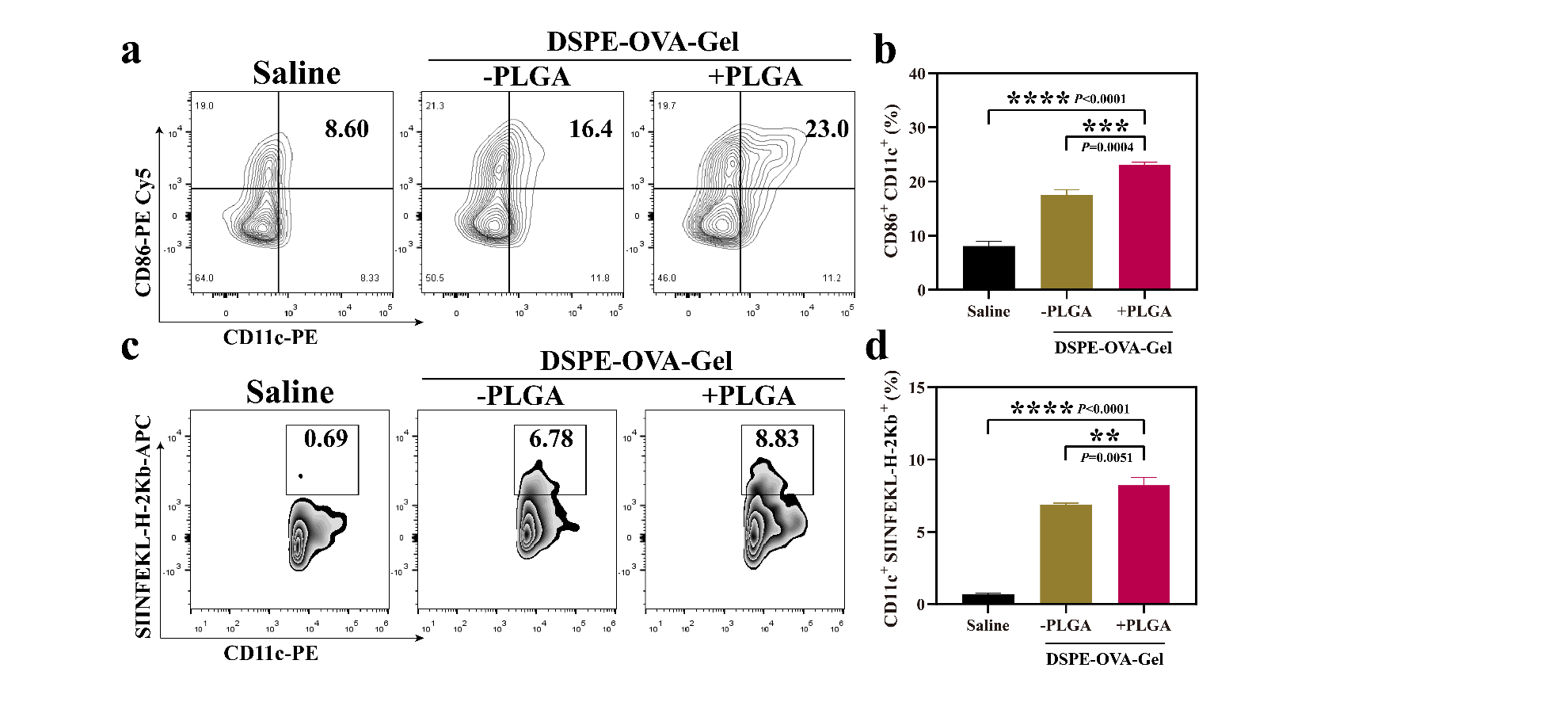


**Figure S13.** Representative flow cytometric analyses of CD11c^+^ CD86^+^ cells (a) and CD11c^+^ SIINFEKL-H-2Kb^+^ cells (c) infiltrated in LNs after receiving different treatments in C57BL/6 mice. The relative quantification of CD11c^+^ CD86^+^ cells (b) and CD11c^+^ SIINFEKL-H-2Kb^+^ cells (d) infiltrated in LNs (n = 3 independent experiments). The results are shown as the mean ± standard deviation. Statistical analyses were performed via ordinary one-way ANOVA with Tukey’s multiple comparisons test, ***p* < 0.01; ****p* < 0.001; *****p* < 0.0001.


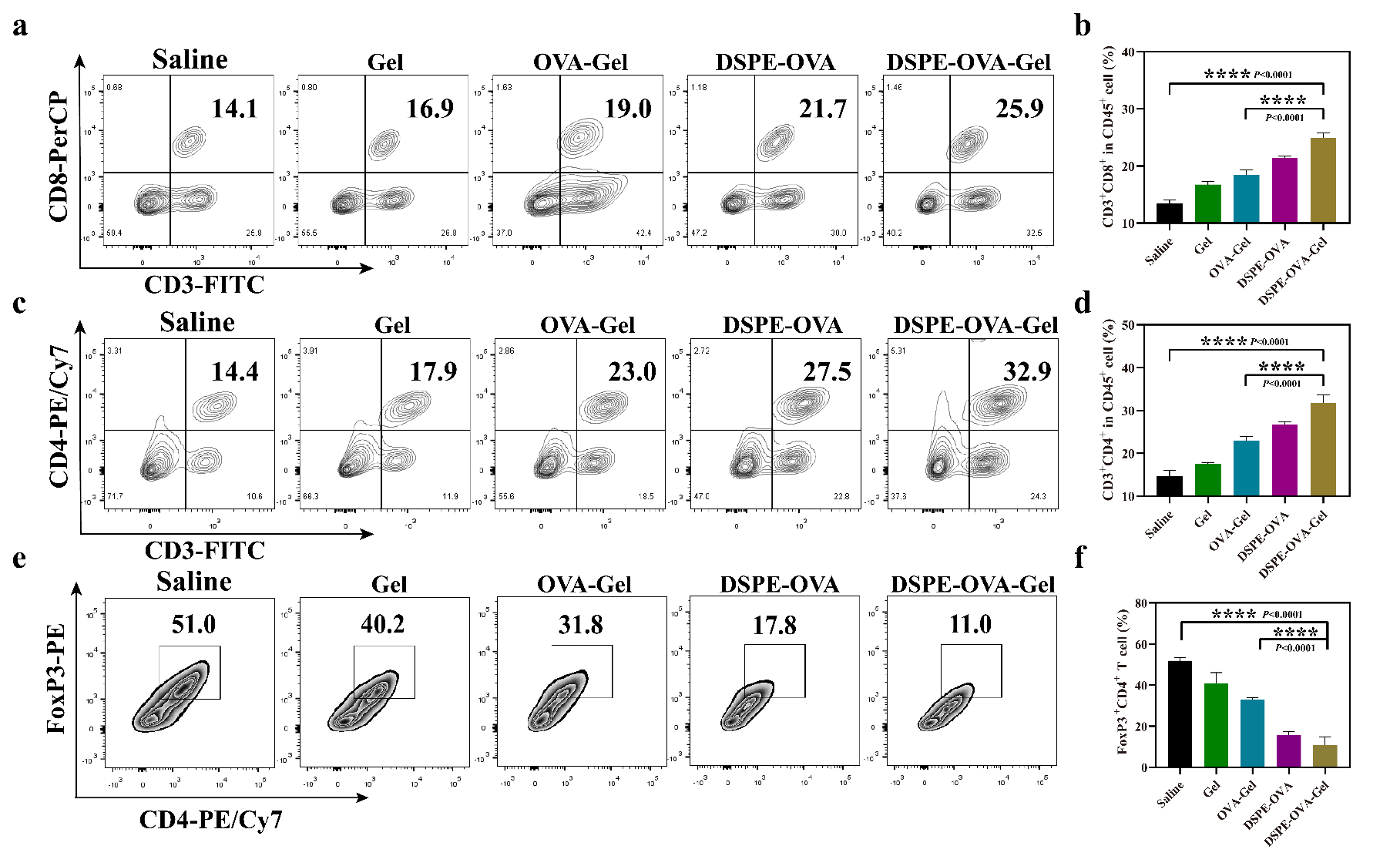


**Figure S14.** Representative flow cytometric analyses of CD3^+^ CD8^+^ T cells (a), CD3^+^ CD4^+^ T cells (c), and Foxp3^+^ CD4^+^ T cells (e) (gate on CD45^+^) infiltrated in tumors after receiving different treatments in C57BL/6 mice. The relative quantification of CD3^+^ CD8^+^ T cells (b), CD3^+^ CD4^+^ T cells (d) and Foxp3^+^ CD4^+^ T cells (f) infiltrated in tumor tissues (n = 3 independent experiments)**.** The results are shown as the mean ± standard deviation. Statistical analyses were performed via ordinary one-way ANOVA with Tukey’s multiple comparisons test, *****p* < 0.0001.


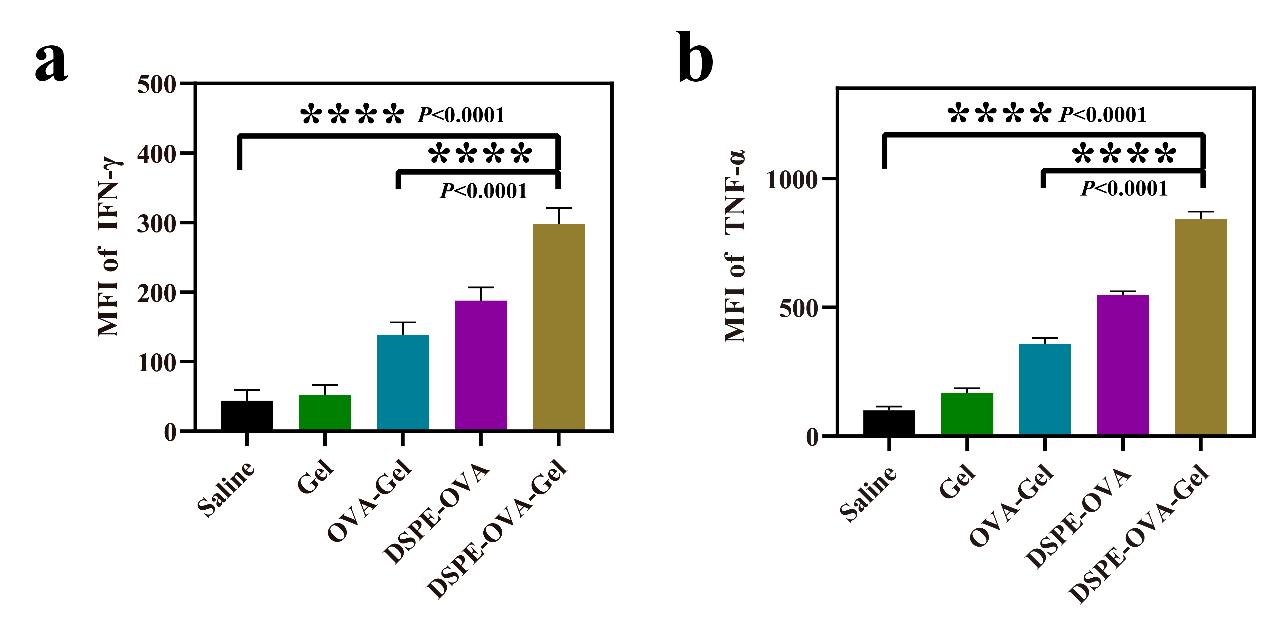


**Figure S15.** The relative quantification of IFN-γ (a) and TNF-α (b) in tumor tissues (n = 3 independent experiments). The results are shown as the mean ± standard deviation. Statistical analyses were performed via ordinary one-way ANOVA with Tukey’s multiple comparisons test, *****p* < 0.0001.


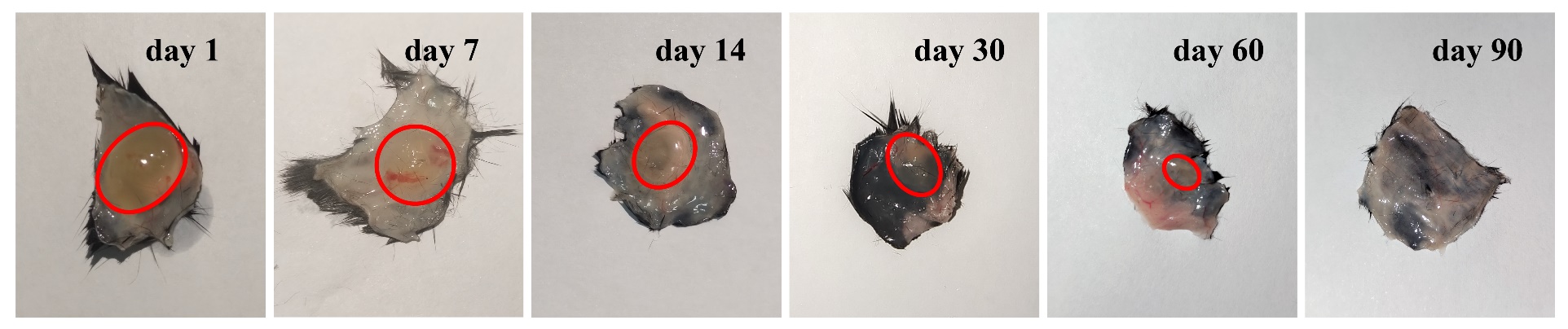


**Figure S16.** The photos of degraded hydrogel under the skin of mice.


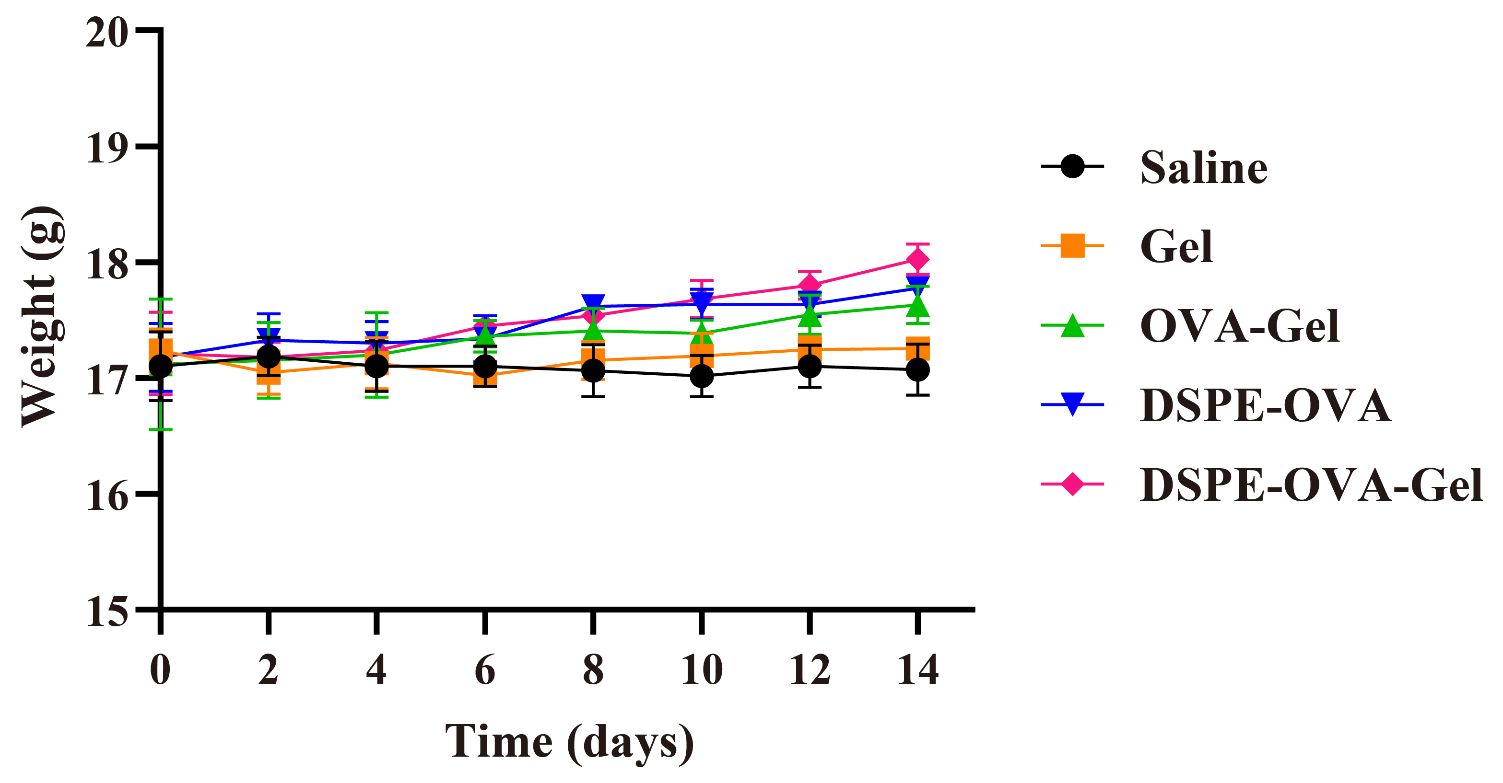


**Figure S17.** Change in body weight of mice in the different groups during the whole animal experiments (n =5 independent experiments).


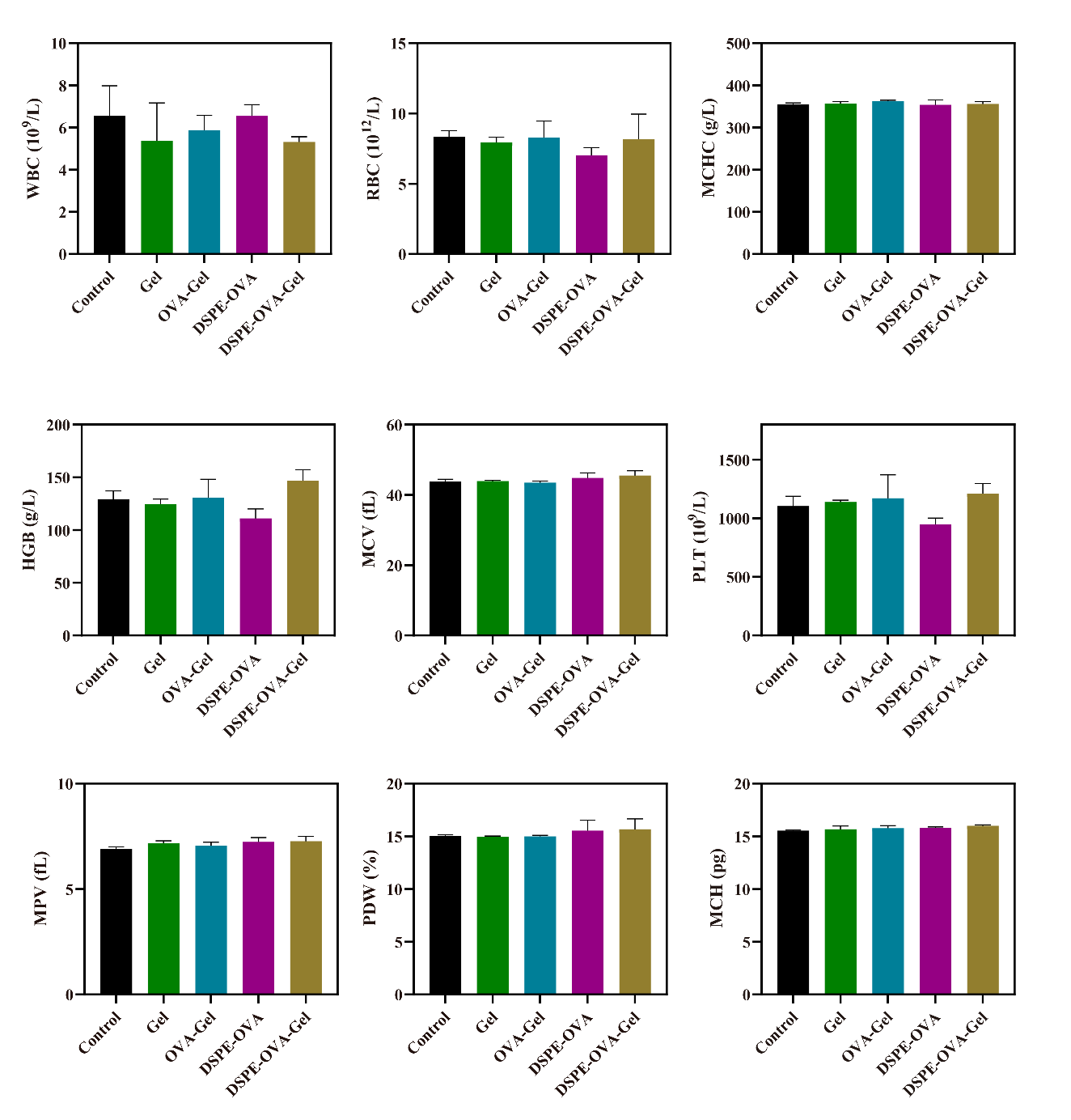


**Figure S18.** The blood routine indicators from mice subjected to different treatments (n = 3 independent experiments). The results are shown as the mean ± standard deviation.


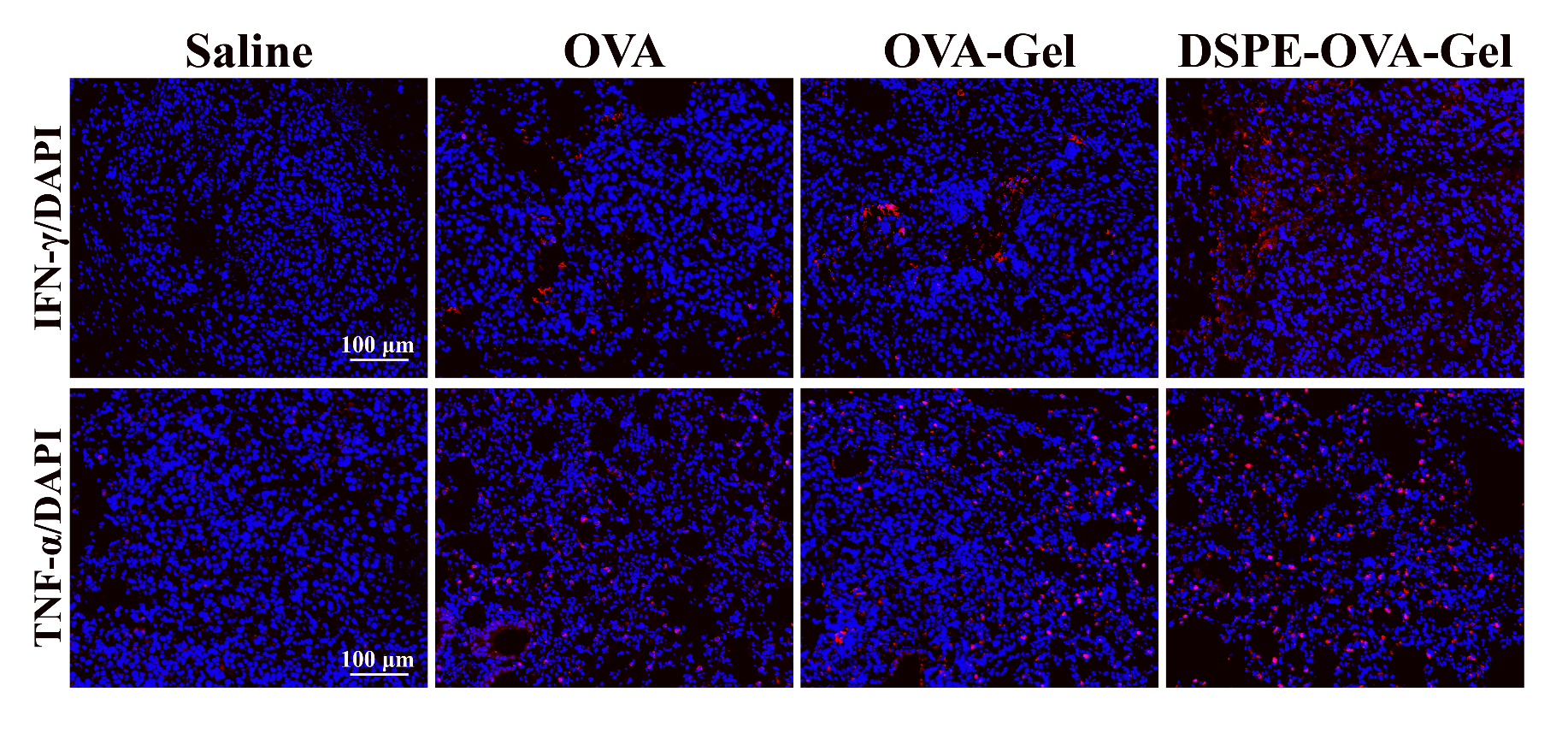


**Figure S19.** Representative immuno-fluorescence staining for IFN-γ and TNF-α in metastasis-bearing lung tissue (IFN-γ/TNF-α: red; Nucleus: blue, scale bar: 100 μm; n = 3 independent experiments).
